# Wingless-related integration site (WNT) signaling is activated during the inflammatory response upon cardiac surgery: A translational study

**DOI:** 10.3389/fcvm.2022.997350

**Published:** 2022-11-11

**Authors:** Bernard D. Krüger, Gilles E. Hofer, Alain Rudiger, Gabriela H. Spahn, Julia Braun, Dominique Bettex, Gabriele Schoedon, Donat R. Spahn

**Affiliations:** ^1^Institute of Anesthesiology, University Hospital Zurich, Zurich, Switzerland; ^2^University of Zurich, Zurich, Switzerland; ^3^Department of Medicine, Limmattal Hospital, Schlieren, Switzerland; ^4^Department of Epidemiology, Epidemiology, Biostatistics and Prevention Institute, University of Zurich, Zurich, Switzerland; ^5^Department of Biostatistics, Epidemiology, Biostatistics and Prevention Institute, University of Zurich, Zurich, Switzerland; ^6^Inflammation Research Unit, Department of Internal Medicine, University Hospital Zurich, University of Zurich, Zurich, Switzerland

**Keywords:** inflammation, cardiac surgery, cardiopulmonary bypass, systemic inflammatory response syndrome, SIRS, WNT signaling, WNT-5a, inflammatory biomarkers

## Abstract

**Objective:**

Cardiac surgery and the use of cardiopulmonary bypass initiate a systemic inflammatory response. Wingless-related integration site (WNT) signaling is part of the innate immunity and has been attributed a major role in the regulation of inflammation. In preclinical research, WNT-5a may sustain an inflammatory response and cause endothelial dysfunction. Our aim was to investigate WNT signaling after cardiac surgery and its association with postoperative inflammation (Clinicaltrials.gov, NCT04058496).

**Methods:**

In this prospective, single-center, observational study, 64 consecutive patients for coronary artery bypass grafting (CABG) ± valve surgery were assigned into three groups: off-pump CABG (*n* = 28), on-pump CABG (*n* = 16) and combined valve-CABG surgery (*n* = 20). Blood samples were acquired before surgery, at intensive care unit (ICU) admission and 4, 8, and 48 h thereafter. Plasma concentrations of WNT-5a and its antagonists Secreted frizzled-related protein 1 (sFRP-1), Secreted frizzled-related protein 5 (sFRP-5), and WNT inhibitory factor 1 (WIF-1) were determined by enzyme-linked immunosorbent assay. In addition, plasma concentrations of six inflammatory cytokines were measured by multiplex immunoassay. Parameters were analyzed for evolution of plasma concentration over time, interactions, intergroup differences, and association with clinical outcome parameters.

**Results:**

At baseline, WNT-5a, sFRP-1, and WIF-1 were present in a minimal concentration, while sFRP-5 was elevated. A higher baseline value of WNT-5a, sFRP-5, and WIF-1 resulted in higher subsequent values of the respective parameter. At ICU admission, WNT-5a and sFRP-5 reached their maximum and minimum value, respectively. WIF-1 decreased over time and was lowest 8 h after surgery. sFRP-1 changed minimally over time. While WNT-5a returned to the baseline within 48 h, sFRP-5 and WIF-1 did not reach their baseline value at 48 h. Of the investigated WNT system components, only WIF-1 partially reflected the severity of surgery. WNT-5a and WIF-1 had an impact on postoperative fluid balance and noradrenaline requirement.

**Conclusion:**

WNT-5a, sFRP-5, and WIF-1 are part of the systemic inflammatory response after cardiac surgery. WNT-5a peaks immediately after cardiac surgery and returns to baseline within 48 h, presumably modulated by its antagonist sFRP-5. Based on this translational study, WNT-5a antagonism may be further investigated to assess potentially beneficial effects in patients with a dysregulated inflammation after cardiac surgery.

## Introduction

During cardiac surgery, a sterile inflammation is initiated by the surgical trauma, blood contact with the artificial surfaces of a cardiopulmonary bypass (CPB) and ischemia-reperfusion injury predominantly of the heart and the lung (1). Damage associated molecular patterns activate the mononuclear phagocyte system, an essential component of the innate immunity, to mount an inflammatory response (2). While a localized inflammation is important for tissue repair and wound healing, a systemic inflammatory response may ensue when cytokines are released into the blood circulation, thereby alerting the jeopardized host and activating defense mechanisms (3). In some patients, the systemic inflammatory response is dysregulated thereby causing a distributive shock which may lead to organ dysfunctions and in the most severe cases even death (4). Microcirculatory dysfunction with vasodilation and vascular leakage are common and clinically important consequences of a systemic inflammation, requiring prompt treatment with vasopressors and intravenous fluids (5).

**FIGURE 1:**
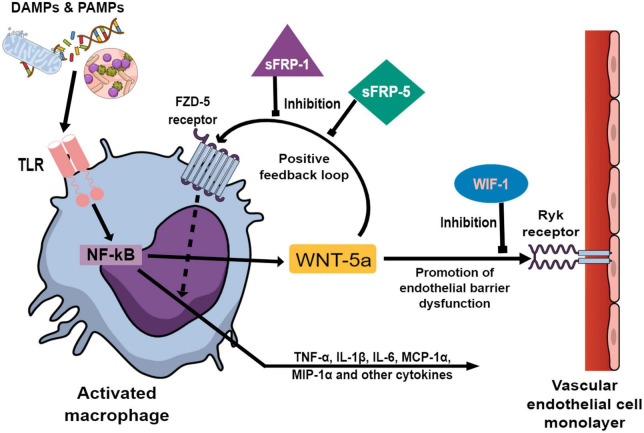
Preclinical studies investigating Wingless-related integration site (WNT) signaling components (9–11, 30) are graphically summarized to elucidate the background of this translational study. Damage and pathogen associated molecular patterns (DAMPs and PAMPs) activate macrophages *via* Toll-like receptors (TLR) which in turn promote transcription factor NF-κB to upregulate the expression of WNT-5a and a wide array of inflammatory cytokines. After secretion, WNT-5a binds to the membrane bound Frizzled-5 (FZD-5) receptor on the activated macrophages leading to an augmentation of the inflammatory cytokine production. This autocrine action enables WNT-5a to sustain the inflammatory response unrelated to the original stimulus. The positive feedback loop of WNT-5a is antagonized by Secreted frizzled-related protein 1 (sFRP-1) and Secreted frizzled-related protein 5 (sFRP-5) at the FZD-5 receptor. In vascular endothelial cells (VEC), WNT-5a promotes the loosening of intercellular tight junctions leading to increased paracellular permeability. This paracrine effect of WNT-5a is antagonized by WNT inhibitory factor 1 (WIF-1) at the membrane bound Ryk-receptor on VEC.

In addition to its involvement in cell development and tissue homeostasis, Wingless-related integration site (WNT) signaling is an integral part of the innate immunity (6). WNT ligands, frizzled receptors and their signaling pathways are profoundly involved in the regulation of proinflammatory mediators and are the subject of ongoing research (7). In particular, WNT-5a may be a valid candidate to readily identify and quantify a systemic inflammation (8). In preclinical research, WNT-5a may sustain the inflammatory response in activated macrophages independently from the original stimulus by a positive feedback loop (9). This autocrine action of WNT-5a is antagonized by the endogenous antagonist Secreted frizzled-related protein 1 (sFRP-1) (10). In cell cultures, WNT-5a diminished the barrier function of vascular endothelial cells (VEC), potentially leading to microvascular leakage. This paracrine action of WNT-5a on VEC is antagonized by WNT inhibitory factor 1 (WIF-1) (11). The preclinical data is graphically summarized in Figure 1. However, it remains unclear if WNT signaling may be utilized to predict and characterize a postoperative systemic inflammation in patients after cardiac surgery.

The aims of this study were (1) to investigate the blood plasma concentration course of the WNT signaling components WNT-5a, sFRP-1, sFRP-5 and WIF-1 after cardiac surgery and to analyze their interaction, (2) to explore the integration of the WNT signaling components during the inflammatory response, and (3) to examine the influence of WNT signaling components on clinical effects associated with postoperative inflammation. Emphasis was given to consider the severity of the surgical trauma and the use of CPB during surgery. We hypothesized that the WNT-5a plasma concentration would increase according to the complexity of cardiac surgery and that a higher WNT-5a plasma value would be associated with a longer period of postoperative hemodynamic instability requiring more vasopressors and a higher fluid administration.

## Materials and methods

The investigation conformed to the principles outlined in the Declaration of Helsinki. Data are reported following the STROBE guidelines (12).

### Study design

The study was designed as a prospective, single-center, observational study. Consecutive patients for coronary artery bypass grafting (CABG) with or without valve surgery were assigned into one of three groups according to the scheduled cardio-surgical procedure: (1) off-pump CABG (OPCAB group), (2) CABG using CPB (on-pump CABG group) and (3) combined CABG and valve surgery (valve-CABG group). It was assumed that the inflammatory response would increase with rising severity of the surgical trauma and the intraoperative use of CPB. In view of the limited pre-existing clinical research investigating WNT signaling in the setting of cardiac surgery, we targeted a sample size of 20 patients per group to reasonably use our resources.

To assess the study’s feasibility, a pilot study comprising ten patients for on-pump CABG surgery was conducted first. We assumed that the on-pump CABG group would produce an intermediate level of systemic inflammation compared to the other groups (13). The study was conducted in the operating theater and the intensive care unit (ICU) for cardiovascular surgery at the University Hospital Zurich, Switzerland. The recruitment period for the pilot study was 06/2018 – 07/2018, and for the main study 11/2018 – 02/2020. Patients were followed up until being discharged from hospital.

**FIGURE 2:**
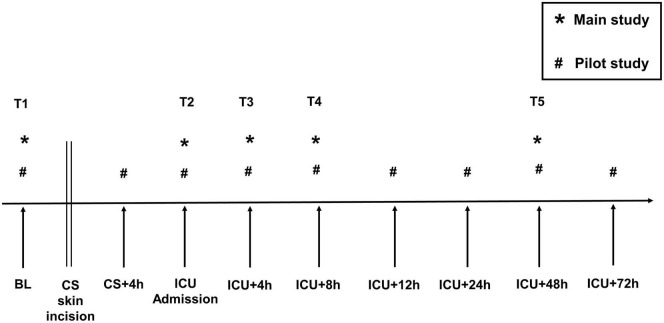
Blood sampling timeline. T1–T5, blood sampling time points used for statistical analysis. BL, Baseline; CS, cardiac surgery; CS + 4 h, 4 h after skin incision; ICU, intensive care unit. *, main study; #, pilot study.

In the pilot study, blood was sampled at nine time points to characterize the inflammatory response over time. Emphasis was placed on the acquisition of a baseline value (T1) before surgery as a reference. After a detailed look at the data acquired in the pilot study, we observed the peak of WNT-5a at the time of ICU admission (T2). At later time points, WNT-5a declined in all pilot study patients. To minimize the amount of blood loss for the patients and to optimize the analysis procedures, we reduced the blood sampling from nine to five time points for the main study. Figure 2 depicts a timeline indicating all blood sampling time points used in the pilot and in the main study.

As the soluble WNT antagonists sFRP-1, sFRP-5 and WIF-1 are considered to bind to WNT-5a forming a complex (10, 11, 14), we assumed these parameters to co-exist in a relationship. To express the balance between WNT-5a and the other WNT parameters as a single variable we calculated ratios between WNT-5a and its antagonists. Ratios were calculated for every patient at all blood sampling time points.

To investigate the role WNT signaling components play in the inflammatory response and to explore potential interactions, we determined the plasma concentrations of inflammatory cytokines. These have been used previously to explore an inflammatory reaction to cardiac surgery (15, 16). In this study, plasma concentrations of interleukin 1β (IL-1β), interleukin 6 (IL-6), tumor necrosis factor α (TNF-α), growth-regulated oncogene α (GRO-α), macrophage inflammatory protein 1α (MIP-1α), and monocyte chemoattractant protein 1α (MCP-1α) were measured together with the WNT signaling components at all time points. More commonly used inflammatory parameters such as the C-reactive protein (CRP) and white cell blood count (WBC) were determined only at the baseline (T1) and at the time of ICU admission (T2) to minimize blood loss for the patients.

An inflammatory response to cardiac surgery may be influenced by additional factors. Blood product exposure has been shown experimentally to act pro-inflammatory in (pre-stimulated) endothelial cells (17). A perioperative glucocorticoid administration may modulate the immune response after cardiac surgery by inducing anti-inflammatory effects (18). To assess an influence of blood product transfusions and steroid use on the change of WNT signaling components upon cardiac surgery, we collected (1) details of red blood cell, platelet, and fresh frozen plasma transfusions intraoperatively and during the first 24 h after surgery and (2) details of a steroid stress dose administration intraoperatively and during the first 8 h after surgery.

A systemic inflammatory response to surgery may cause an endothelial dysfunction leading to a microvascular leakage and a reduced vasomotor tone potentially culminating in a distributive shock (19). In this study, we used three clinical outcome parameters as surrogates to quantify the clinical effects of a postoperative inflammation in our patients: (1) the fluid balance of the ICU admission day, (2) the noradrenaline dosage at the time of ICU admission, and (3) the time taken for noradrenaline to decline below <0.1 μg/kg/min with no concomitant inotropic drug therapy after ICU admission, which was defined as the “duration of hemodynamic instability.”

### Participants

Inclusion criteria were age >18 years, cardiac surgery *via* sternotomy, CABG with or without valve surgery and postoperative admittance to the cardio-surgical ICU. Exclusion criteria were the presence of preoperative infection, preexisting immunosuppressive therapy and the withdrawal of informed consent. Patients requiring a mass transfusion (>10 red blood cell concentrates within 24 h) and/or extracorporeal life support after cardiac surgery were excluded from the analysis.

### Blood sample acquisition, handling, storage, and processing for laboratory analysis

Particular care was taken to keep the time interval between the acquisition and the laboratory analysis of the blood samples as short as possible. At each time point (T1–T9), venous blood was collected in a ethylenediaminetetraacetic acid (EDTA) containing vacutainer. Every blood sample was promptly delivered to the in-hospital laboratory of the Institute of Clinical Chemistry for timely processing. The blood samples were centrifuged at 15,000 rpm for 3 min and the resulting plasma aliquots were filled into microtubes, frozen and stored at −20°C for up to 3 days. Thereafter, all samples were transferred frozen for long term storage at −80°C. An analysis of blood samples was subsequently performed when samples of a batch of up to 16 patients were acquired. For laboratory analysis the aliquots were thawed at 4°C. The blood plasma was centrifuged at 15,000 rpm for 5 min to separate the plasma from any residual sediment. During the preparation of the laboratory kits the aliquots were kept at maximum 4°C. Freeze-thaw cycles were avoided.

Blood samples were blinded for laboratory analysis. The laboratory kits were ordered from the manufacturer shortly before being used to avoid long-term storage associated quality issues. No cooling chain interruption occurred. All laboratory analyses were conducted in accordance with the manufacturer’s recommendations. Free plasma concentrations of WNT-5a, sFRP-1, sFRP-5, and WIF-1 were determined by enzyme-linked immunosorbent assay (LifeSpan BioSciences, Inc., Seattle, WA, USA). Free plasma concentrations of IL-1β, IL-6, TNF-α, GRO-α, MIP-1α, and MCP-1α were measured by multiplex immunoassay (Bio-Plex Pro Human Inflammation Assays 6-plex and Biorad 200 Reader, Bio-Rad Laboratories AG, Cressier, Switzerland). Sensitivity levels were as follows: WNT-5a: 0.094 ng/ml, sFRP-1: 0.094 ng/ml, sFRP-5: 0.64 ng/ml, WIF-1: 5.9 pg/ml, IL-1β: 0.24 pg/ml, IL-6: 0.34 pg/ml, TNF-α: 1.13 pg/ml, GRO-α: 13.45 pg/ml, MIP-1α: 0.06 pg/ml, and MCP-1α: 0.44 pg/ml.

### Perioperative patient management

After the application of standard monitoring, the patients were anesthetized for surgery. General anesthesia was induced with propofol and maintained with propofol or sevoflurane. Fentanyl and continuous sufentanil were used for analgesia and rocuronium for muscle relaxation. Perioperative cardiac function was evaluated by transesophageal echocardiography. Cardiac surgery was performed *via* median sternotomy. For cardiac bypass grafting, OPCAB patients received an initial heparin bolus of 250 IU/kg iv followed by the repeated administration of heparin 5,000 IU iv to obtain an activated clotting time of >350 s. Before CPB commencement for on-pump CABG with or without valve surgery, patients received heparin 300 IU/kg plus tranexamic acid 15 mg/kg iv followed by the repeated administration of heparin 5,000 IU iv to establish an activated clotting time of >500 s. The CPB system (Stöckert S5, LivaNova, UK; CAPIOX^®^ FX25-Oxygenator, Terumo, Japan) was equipped with heparin coated tubes and primed with Ringerfundin^®^, tranexamic acid 500 mg and heparin 10,000 IU. During CPB, a blood flow according to a cardiac index of 2.4 l/min/m^2^ was targeted. The patient’s body temperature was lowered as specified by the attending surgeon. After completion of cardiac bypass grafting in OPCAB patients or weaning from CPB in patients receiving on-pump CABG with or without valve surgery, the initial heparin bolus was antagonized with protamine 1:1 iv. Blood product transfusion and treatment of coagulopathy were guided according to published guidelines of the University Hospital Zurich, Switzerland (20). An autologous retransfusion device (Cell Saver^®^ Elite^®^, Haemonetics S.A., Signy Centre, Switzerland) was used to reinfuse shed mediastinal blood and residual blood from the CPB circuit. Postoperatively, all patients were admitted to the cardio-surgical ICU. Treatment guidelines for this ICU have been described earlier (21, 22). Patients were continuously assessed for shock and occurrence of postoperative complications. Sedation and mechanical ventilation were discontinued in all patients as soon as possible.

The administration of intravenous fluids and the use of vasoactive drugs were at the discretion of the attending anesthesiologist or intensivist. Intravascular volume was restored by infusion of balanced crystalloid and/or colloid solutions (Ringerfundin^®^ and Physiogel^®^, respectively; B. Braun Medical AG, Sempach, CH). In patients with renal insufficiency albumin 5% (CSL Behring AG, Bern, Switzerland) was used as colloidal fluid replacement in the ICU. Noradrenaline was used as the first line vasopressor drug. If the noradrenaline dosage exceeded 0.3 μg/kg/min perioperatively, an empirical steroid stress dose of hydrocortisone was administered with an initial bolus of 100 mg iv followed by 50 mg iv qid. Hydrocortisone treatment was tapered off within 5 days.

### Data sources and database

Clinical data were collected from the hospital’s electronic patient data management systems KISIM (CISTEC AG, Zürich, Switzerland) and Metavision (iMDsoft^®^, Tel Aviv, Israel). All clinical and laboratory data were entered in a web based, good clinical practice compliant electronic data capture system (secuTrial^®^, interActive Systems GmbH, Berlin, Germany). The database was closed in 09/2020.

### Statistical analysis

Statistical analyses were conducted using R (Version 4.0.5). Descriptive statistics are given as numbers (percentages) for categorical data and as median (interquartile range) or mean (±SD) for continuous data. The effect of cardiac surgery on WNT signaling components and inflammatory cytokines was investigated firstly by combining all surgical procedures and secondly by dividing the population into the three groups with increasing surgical complexity.

Differences of WNT signaling components and inflammatory cytokines between the baseline (T1) and the time of ICU admission (T2) were analyzed using Wilcoxon tests for paired data. Note that we performed Wilcoxon tests for paired data only for the total dataset and stuck to a descriptive comparison per group to avoid a multiple testing problem. To further analyze the data, linear models were calculated to analyze data at a single time point, and linear mixed models with random intercept per patient to analyze repeated measurements over time. We did not analyze sFRP-1 in depth because this parameter changed only minimally over time.

Intergroup differences of WNT signaling components and inflammatory cytokines between the OPCAB, on-pump CABG and valve-CABG groups were investigated stepwise. The OPCAB group was used as the reference group. In a first step, differences between the three groups at the time of ICU admission (T2) were evaluated using a linear model. In a second step, differences over all time points after surgery (T2–T5) were examined using a linear mixed model adjusted for the respective baseline value (T1) and the respective time point. The different time points were included as a categorical variable to capture a possibly non-linear behavior of the outcome variables over time. We did not adjust for the severity of surgery (e.g., its duration) because this was covered by dividing patients into the three groups. Adding a surgery related variable would lead to multicollinearity and render the models non-interpretable.

To explore the event of a blood product transfusion or a perioperative steroid stress dose administration as potential confounders, these parameters were included in the mixed models for the WNT signaling components as a sensitivity analysis. Concerning blood products, only the event of an intraoperative transfusion was used to account for the temporal relationship between cause and effect.

Relations between WNT signaling components and inflammatory cytokines were explored stepwise. First, Spearman’s rank correlation coefficient was calculated at the baseline (T1), the time of ICU admission (T2) and 48 h after surgery (T5). We chose this correlation coefficient because it is not sensitive to outliers. Second, the influence of WNT-5a on other WNT parameters and inflammatory cytokines over all time points (T1–T5) was assessed using linear mixed models.

The influence of WNT signaling components and inflammatory cytokines at the time of ICU admission (T2) on the three clinical outcome parameters (1) fluid balance of the ICU admission day, (2) norepinephrine dosage at the time of ICU admission (T2), and (3) duration of hemodynamic instability was explored using linear models. As covariates the values of WNT-5a, sFRP-5, WIF-1, IL-6, and MCP-1α at T2 were used.

### Handling of missing data

Only in one patient of the valve-CABG group, data from blood sampling at one single time point, 48 h after surgery (T5), were missing. This was not expected to change the results. In the linear models we compared only values at the time of ICU admission (T2) for which the dataset was complete. The linear mixed models applied to analyze data over multiple time points used all available values without excluding a whole patient because of missing values. For these reasons and because of the otherwise complete dataset, the missing data did not necessitate any action in this study.

### Presentation of data

Box plots show the box with the median, representing 25th, 50th, and 75th percentiles. Their whiskers represent values within 1.5× IQR. Values outside this range are outliers and extremes. WNT-5a, sFRP-5, and IL-6 are presented in figures with a zoomed scale to enable a reasonable graphical presentation. To comprehensively report our data, values of WNT signaling components including ratios, and inflammatory cytokines at all time points are presented in detail as Supplementary material.

## Results

### Patient population, surgery, and clinical outcome

A total of 73 consecutive patients were assigned to one of the three groups. Of these, nine patients were excluded because of cancellation of surgery (*n* = 2), study consent withdrawal (*n* = 1), a preoperative cardiac event with ICU admission (*n* = 1), pre-existing immunosuppressive therapy (*n* = 1), ECLS implantation (*n* = 2), and erroneous blood sampling (*n* = 2). Finally, 64 patients were included in the analysis. Follow-up was completed in 100% of included patients. Median follow-up time was 8 (7–12) days.

Patient and surgery characteristics are presented in Table 1. As four patients of the on-pump group were operated in off-pump technique after group assignment, the number of patients differed between the three groups. Patients had a median age of 70 years, were predominantly male and pre-obese. Approximately half of the patients were active or former smokers. Two-thirds of the operations were elective. The valve-CABG group included patients with aortic valve replacement (*n* = 13) or reconstruction (*n* = 2), mitral valve replacement (*n* = 2) or reconstruction (*n* = 5) and tricuspid valve reconstruction (*n* = 2). Three patients had interventions in up to three valves, and two patients underwent isolated valve surgery. Overall, a median of 3 (2–4) bypass grafts were performed. The highest intraoperative fluid administration was found in the OPCAB group. The three groups differed considering the type and the duration of surgery, use of CPB and aortic cross clamp time, with highest values in the valve-CABG group, intermediate values in the on-pump CABG group and lowest values in the OPCAB group. Overall, the lowest body temperature during surgery was 35.0 (34.0–35.6) °C.

**TABLE 1:** Patient characteristics and details of cardiac surgery.

	Total (*n* = 64)	OPCAB (*n* = 28)	On-pump CABG (*n* = 16)	Valve-CABG (*n* = 20)
**Patient population**				
Age (years)	70 (62–74)	70 (62–77)	69 (61–72)	71 (67–76)
Male gender (*n*)	51 (80%)	24 (86%)	11 (68%)	16 (80%)
Body mass index (kg/m^2^)	27 (25–31)	27 (26–30)	27 (25–31)	27 (23–30)
**Tobacco smoking**				
Pack years (years)	4 (0–34) *n* = 34	23 (0–45) *n* = 19	0.5 (0–36) *n* = 8	0 (0–6) *n* = 7
**Preoperative blood laboratory values**				
Hemoglobin (g/L)	140 (131–149)	140 (134–151)	141 (126–148)	141 (134–149)
Platelet count (g/L)	223 (179–258)	224 (182–255)	223 (190–266)	218 (173–265)
hsTroponin (ng/L)	14 (8–27)	14 (8–34)	17 (12–39)	13 (8–17)
Creatinine (μmol/L)	84 (72–98)	89 (71–99)	80 (72–93)	83 (77–93)
**Cardiac surgery details**				
EuroSCORE II (points)	1.9 (1.1–2.9)	1.2 (0.9–2.2)	1.9 (1.1–3.8)	2.8 (1.9–4.0)
Type of surgery				
Elective (*n*)	43 (67%)	16 (57%)	10 (63%)	17 (85%)
Urgent (*n*)	21 (33%)	12 (43%)	6 (38%)	3 (15%)
Surgery duration (min)	270 (237–326)	254 (200–276)	257 (245–298)	321 (265–360)
CPB duration (min)	97 (0–138)	n.a.	115 (105–123)	153 (136–185)
Aortic cross clamp duration (min)	61 (0–95)	n.a.	71 (61–82)	111 (92–135)
Bypass grafts (*n*)	3 (2–4)	3 (3–4)	3 (3–4)	2 (1–3)
Lowest body temperature (°C)	35.0 (34.0–35.6)	35.6 (35.2–36.3)	34.7 (34.0–35.3)	33.8 (32.7–34.2)
Intraoperative fluid administration (L)	4.2 (2.6–5.4)	5.1 (4.1–6.6)	2.9 (2.5–3.3)	3.3 (2.3–4.6)
**Echocardiography**				
Preoperative LVEF (%)	60 (51–62)	55 (50–60)	57 (50–61)	60 (55–65)
Postoperative LVEF (%)	55 (50–65)	55 (50–61)	58 (50–65)	58 (49–65)
**Intraoperative blood product transfusions**				
Patients with RBC transfusions (*n*)	6 (9%)	1 (4%)	1 (6%)	4 (20%)
RBC units (*n*)	0.2 (±0.7)	0.1 (±0.8)	0.2 (±0.8)	0.2 (±0.4)
Patients with platelet transfusions (*n*)	11 (17%)	3 (11%)	2 (13%)	6 (30%)
Platelet units (*n*)	0.2 (±0.5)	0.1 (±0.3)	0.2 (±0.5)	0.4 (±0.7)
**Intraoperative steroid stress dose administration**				
Hydrocortisone (*n*)	7 (11%)	2 (7%)	2 (13%)	3 (15%)

CABG, coronary artery bypass grafting; CBP, cardiopulmonary bypass; CRP, C-reactive protein; LVEF, left ventricular ejection fraction; n.a., not applicable; OPCAB, off-pump coronary artery bypass grafting; RBC, red blood cells. Values are presented as number (percentage), median (interquartile range), or mean (±SD).

Clinical outcome data are presented in Table 2. ICU and hospital survival were 100 and 97%, respectively. Two patients in the on-pump CABG group died postoperatively while in the hospital. One of these patients suffered a major cerebral stroke after cardiac surgery and died on postoperative day 20. The other patient died after an in-hospital cardiac arrest on postoperative day 3. Patients of the valve-CABG group tended to stay longer in the ICU, although the length of ICU stay was overall short. Duration of hemodynamic instability and daily fluid balance were highest in the valve-CABG group, intermediate in the on-pump CABG group and lowest in the OPCAB group. The highest noradrenaline dosage in the ICU was observed in the valve-CABG group, while the other groups had comparable values. Perioperatively, hydrocortisone was administered empirically as a steroid stress dose treatment in a total of 11 (17%) patients. One of these patients received hydrocortisone as a onetime bolus only during surgery and in four patients, hydrocortisone treatment was commenced within 8 h after surgery. Tables 1, 2 summarize patients with a steroid stress dose treatment according to the timing of hydrocortisone administration. A pneumonia was postulated in three patients 24 h, 48 h and 6 days after surgery, respectively. In these patients, an empirical antibiotic therapy with piperacillin–tazobactam was initiated. Perioperatively, blood products were administered in a total of 21 (33%) patients. Red blood cells were transfused in 18 (28%) patients and platelets in 13 (20%) patients. Regarding the three groups, a transfusion of red blood cells and platelets occurred mostly in patients in the valve-CABG group (*n* = 7, 35%, for both blood products), followed by the OPCAB group (*n* = 8, 29% and *n* = 4, 14%, respectively) and the on pump-CABG group (*n* = 3, 19% and *n* = 2, 13%, respectively). A transfusion of fresh frozen plasma occurred only postoperatively in 3 (15%) patients in the valve-CABG group. Tables 1, 2 summarize the details of blood product transfusions according to the timing of the administration.

**TABLE 2:** Intensive care unit parameters and patient outcome.

	Total (*n* = 64)	OPCAB (*n* = 28)	On-pump CABG (*n* = 16)	Valve-CABG (*n* = 20)
**Length of stay and patient survival**				
Length of ICU stay (days)	1 (1–2)	1 (1–1)	1 (1–2)	2 (1–4)
Length of hospital stay (days)	8 (7–12)	7 (7–11)	8 (7–12)	9 (8–13)
Survival ICU (*n*)	64 (100%)	28 (100%)	16 (100%)	20 (100%)
Survival hospital (*n*)	62 (97%)	28 (100%)	14 (88%)	20 (100%)
**ICU scores**				
SAPS II (points)	28 (24–34)	28 (26–33)	26 (24–28)	32 (26–35)
SOFA ICU admission day (points)	6 (6–8)	6 (6–7)	6 (5–8)	8 (6–9)
SOFA POD 1 (points)	7 (4–8) *n* = 23	6 (4–8) *n* = 7	7 (5–8) *n* = 5	7 (5–9) *n* = 11
**Neurologic system**				
Postoperative delirium (*n*)	11 (17%)	2 (7%)	3 (19%)	6 (30%)
**Blood laboratory values at ICU admission**				
Hemoglobin (g/L)	112 (95–124)	115 (102–131)	104 (93–120)	105 (94–121)
Platelet count (G/L)	137 (110–172)	147 (122–178)	148 (118–176)	115 (80–140)
hsTroponin (ng/L)	566 (297–1,635)	289 (212–434)	840 (489–1,083)	2,415 (1,569–3,956)
Creatinine (μmol/L)	77 (59–86)	80 (66–89)	76 (57–84)	77 (63–84)
**Cardiovascular system**				
Duration of hemodynamic instability^1^ (h)	9 (0–19)	6 (0–16)	12 (0–19)	15 (5–35)
**Drug support at ICU admission**				
Norepinephrine (*n*)	63 (98%)	27 (96%)	16 (100%)	20 (100%)
Norepinephrine dose (μg/min)	8 (5–14)	8 (5–14)	7 (5–10)	10 (7–16)
Inotropes (*n*)	13 (20%)	3 (11%)	4 (25%)	6 (30%)
**Drug support at ICU admission + 8 h**				
Norepinephrine (*n*)	50 (78%)	20 (71%)	11 (69%)	19 (95%)
Norepinephrine dose (μg/min)	5 (1–12)	4 (0–12)	3 (0–11)	7 (4–11)
Inotropes (*n*)	12 (19%)	3 (11%)	4 (25%)	5 (25%)
**Postoperative steroid stress dose administration (0–8 h of ICU)**				
Hydrocortisone (*n*)	10 (16%)	4 (14%)	2 (13%)	4 (20%)
**Fluid balance**				
ICU admission day (L)	2.9 (1.9–4.4)	2.5 (1.9–4.0)	2.8 (2.0–4.4)	3.4 (2.2–4.9)
Postoperative day 1 (L)	0.7 (–0.2 to 1.5) *n* = 24	0.5 (–1.5 to 0.8) *n* = 8	1.1 (0.8–1.3) *n* = 5	0.5 (–0.4 to 2.3) *n* = 11
**Postoperative blood product transfusions (0–24 h of ICU)**				
Patients with RBC transfusions (*n*)	14 (22%)	7 (25%)	3 (19%)	4 (20%)
RBC units (*n*)	0.5 (±1.4)	0.3 (±0.5)	0.3 (±0.8)	0.8 (±2.3)
Patients with FFP transfusions (*n*)	3 (5%)	0 (0%)	0 (0%)	3 (15%)
FFP units (*n*)	0.2 (±1.2)	0 (±0)	0 (±0)	0.8 (±2)
Patients with platelet transfusions (*n*)	6 (9%)	1 (4%)	1 (6%)	4 (20%)
Platelet units (*n*)	0.2 (±0.7)	0 (±0.2)	0.1 (±0.2)	0.4 (±1.1)

FFP, fresh frozen plasma; ICU, intensive care unit; RBC, red blood cells; SAPS, simplified acute physiology score; SOFA, sequential organ failure assessment; POD, postoperative day. “^1^” defined as time from ICU admission until iv norepinephrine dosage <0.1 μg/kg/min and no inotropic drug therapy. Values are presented as number (percentage), median (interquartile range), or mean (±SD).

### Wingless-related integration site signaling components

**FIGURE 3:**
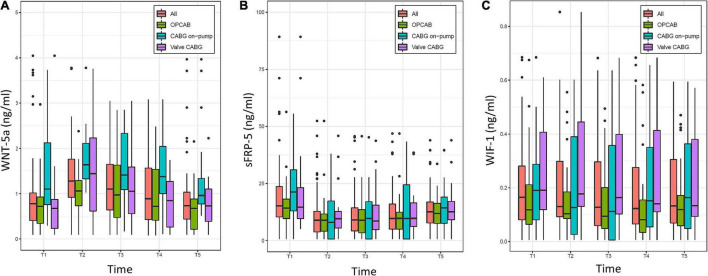
Evolution of **(A)** Wingless-related integration site 5a (WNT-5a), **(B)** Secreted frizzled-related protein 5 (sFRP-5), and **(C)** WNT inhibitory factor 1 (WIF-1) over time (T1–T5) in the overall population (All) and the three groups (OPCAB, CABG on-pump, and valve-CABG). Values are presented as median and interquartile range (IQR) (box), within 1.5× IQR (line) and outliers (dots). T1, baseline; T2, time of intensive care unit (ICU) admission; T3, 4 h after surgery; T4, 8 h after surgery; T5, 48 h after surgery; CABG, coronary artery bypass grafting; OPCAB, off-pump coronary artery bypass grafting.

The evolution of WNT-5a, sFRP-5, and WIF-1 over time is displayed in Figure 3. For the overall population, WNT-5a, sFRP-1, and WIF-1 were present in a minimal concentration at baseline, while sFRP-5 was elevated. WNT-5a was highest at the time of ICU admission and thereafter progressively returned to baseline within 48 h. sFRP-5 was lowest at the time of ICU admission and, rising thereafter but without reaching the baseline value at 48 h after surgery. WIF-1 showed a low kinetic profile and was lowest at 8 h after surgery. sFRP-1 changed only minimally over time in all samples throughout the study (not shown).

The baseline (T1) and time of ICU admission (T2) values of the WNT signaling components are presented in Table 3. For the overall population, we found very strong evidence for a difference in all parameters except for WIF-1 when comparing T2 to T1. A higher median value of WNT-5a, and a lower median value of sFRP-5 were found, whereas the change in sFRP-1 was (albeit statistically significant) numerically minimal. According to a linear mixed model, for every ng/ml (for WNT-5a and sFRP-5) or pg/ml (for WIF-1) increase at T1 the estimated values of T2–T5 increased for WNT-5a by 0.53 ng/ml (0.47–0.58, *p* < 0.0001), for sFRP-5 by 0.57 ng/ml (0.43–0.71, *p* < 0.0001) and for WIF-1 by 0.9 pg/ml (0.79–1.0, *p* < 0.0001), respectively.

**TABLE 3:** Baseline (T1) and ICU admission values (T2) of Wingless-related integration site (WNT) signaling components, cytokines, and inflammatory parameters.

Parameter	Total (*n* = 64)			OPCAB (*n* = 28)		On-pump CABG (*n* = 16)		Valve-CABG (*n* = 20)	
	T1	T2	*P*-value	T1	T2	T1	T2	T1	T2
WNT-5a (ng/ml)	0.78 (0.43–1.1)	1.3 (0.93–1.9)	*p* < 0.0001	0.75 (0.36–0.98)	1.1 (0.74–1.3)	1.1 (0.76–2.1)	1.7 (1.4–2.2)	0.7 (0.27–0.90)	1.5 (0.65–2.3)
sFRP-1 (ng/ml)	0.09 (0.09–0.48)	0.09 (0.09–0.09)	*p* = 0.007	0.09 (0.09–0.09)	0.09 (0.09–0.09)	0.09 (0.09–1.1)	0.09 (0.09–0.17)	0.09 (0.09–6.2)	0.09 (0.09–0.26)
sFRP-5 (ng/ml)	15.2 (10.3–23.6)	8.9 (3.8–12.8)	*p* < 0.0001	14.3 (9.7–18.2)	8.9 (4.1–11.6)	21.3 (13–31)	8.0 (0.6–17.3)	14.6 (9.6–23.1)	9.6 (5.5–12.8)
WIF-1 (ng/ml)	0.17 (0.08–0.28)	0.13 (0.09–0.3)	*p* = 0.84	0.12 (0.06–0.21)	0.10 (0.09–0.18)	0.19 (0.08–0.29)	0.13 (0.03–0.39)	0.19 (0.12–0.41)	0.18 (0.13–0.44)
Ratio WNT-5a/sFRP-5	0.04 (0.02–0.07)	0.14 (0.10–0.26)	*p* < 0.0001	0.03 (0.02–0.08)	0.13 (0.08–0.17)	0.05 (0.03–0.10)	0.19 (0.03–1.9)	0.03 (0.00–0.06)	0.15 (0.02–0.23)
Ratio WNT-5a/WIF-1	3.4 (1.5–18.2)	8.4 (3.2–22.6)	*p* = 0.004	5.2 (1.7–34.1)	10.5 (0.69–22.9)	4.9 (3.1–33.4)	14.5 (4.4–83.0)	1.8 (1.5–3.3)	4.3 (2.9–8.8)
IL-6 (ng/ml)	0.00 (0.00–0.00)	0.29 (0.14–0.66)	*p* < 0.0001	0.00 (0.00–0.00)	0.15 (0.11–0.33)	0.00 (0.00–0.00)	0.29 (0.15–0.4)	0.00 (0.00–0.00)	0.76 (0.33–1.3)
MCP-1α (ng/ml)	0.04 (0.02–0.05)	0.50 (0.28–1.0)	*p* < 0.0001	0.03 (0.03–0.05)	0.34 (0.21–0.64)	0.03 (0.02–0.04)	0.55 (0.29–1.2)	0.04 (0.03–0.05)	1.1 (0.74–1.5)
CRP (mg/L)	3 (1–5)	2 (1–4)	*p* = 0.045	3 (1–6)	2 (1–4)	4 (1–6)	2 (1–4)	2 (1–4)	1 (1–4)
White blood cell count (g/L)	7.1 (5.6–8.2)	12.6 (10.4–15.8)	*p* < 0.0001	7.7 (6.5–8.4)	12.6 (11.5–17.0)	6.8 (5.8–7.6)	13.1 (8.2–15.1)	6.3 (5.4–7.6)	12.6 (10.1–14.2)

T1, baseline; T2, ICU admission; CABG, coronary artery bypass grafting; CRP, C-reactive protein; IL, interleukin; MCP, monocyte chemoattractant protein; OPCAB, off-pump coronary artery bypass grafting; sFRP, secreted frizzled-related protein; WNT, wingless-related integration site; WIF, WNT inhibitory factor. Data are presented as median (interquartile range). For statistical analysis Wilcoxon tests for paired data were used.

The sensitivity analysis for the event of a perioperative steroid stress dose administration, and the event of an intraoperative transfusion of either red blood cell concentrates, or platelets did not change the mixed model estimates for WNT-5a, sFRP-5, and WIF-1.

Regarding intergroup differences, at T2 moderate evidence was found for WIF-1 to be on average 121 pg/ml (11–230, *p* = 0.031) higher in the valve-CABG group compared to the OPCAB group using a linear model. Over all time points after surgery (T2–T4), no evidence for an intergroup difference for any WNT signaling component was found in the linear mixed model.

With respect to the interaction between the WNT signaling components, Spearman’s correlation at T1, T2, and T5 showed low coefficients between WNT-5a and sFRP-5 (0.19, 0.43, and 0.3, respectively), and WNT-5a and WIF-1 (0.15, 0.25, and 0.22, respectively). Accordingly, no evidence for an association between the WNT signaling components was found using a linear mixed model.

**FIGURE 4:**
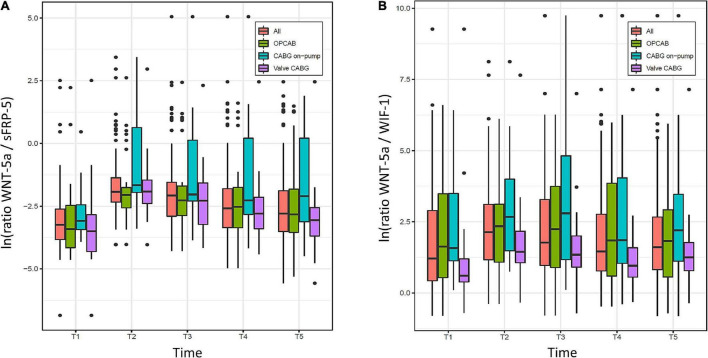
Evolution of ratios between **(A)** Wingless-related integration site 5a (WNT-5a) and Secreted frizzled-related protein 5 (sFRP-5), and **(B)** WNT-5a and WNT inhibitory factor 1 (WIF-1) over time (T1–T5) in the overall population (All) and the three groups (OPCAB, CABG on-pump and valve-CABG). Values are presented as median and interquartile range (IQR) (box), within 1.5× IQR (line) and outliers (dots). ln, natural logarithm; T1, baseline; T2, time of intensive care unit (ICU) admission; T3, 4 h after surgery; T4, 8 h after surgery; T5, 48 h after surgery; CABG, coronary artery bypass grafting; OPCAB, off-pump coronary artery bypass grafting.

The evolution of the ratios between WNT-5a and sFRP5, and between WNT-5a and WIF-1 over time is displayed in Figure 4. For the overall population, the lowest median value of both ratios was found at the baseline (T1) and the highest median value at the time of ICU admission (T2). Thereafter, WNT-5a/sFRP-5 progressively declined but did not reach the baseline value within 48 h. WNT-5a/WIF-1 declined until 8 h after surgery (T4) and then tended to increase again at 48 h after surgery (T5). Using Wilcoxon tests for paired data, the median values of both ratios were higher at T2 compared to T1 (Table 3). No evidence for an intergroup difference was found for both ratios at T2 nor over all time points after surgery (T2–T5).

### Cytokines and inflammatory parameters

For all investigated inflammatory cytokines a low baseline value was found. IL-1β was mostly below the detection limit at all time points (data not shown). For the overall population, TNF-α, GRO-α, and MIP-1α showed a kinetic profile with very low values over time.

**FIGURE 5:**
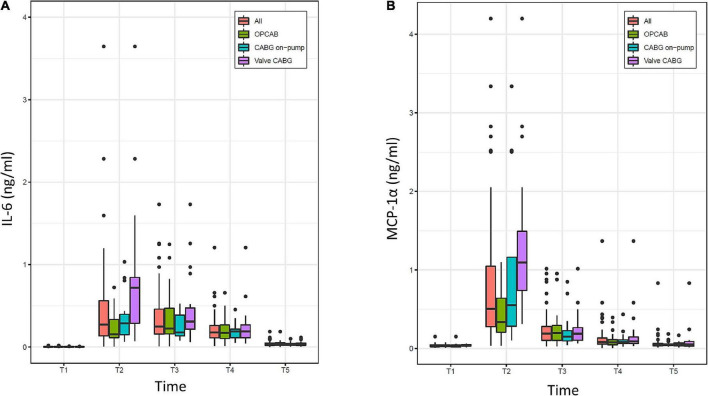
Evolution of **(A)** interleukin-6 (IL-6) and **(B)** monocyte chemoattractant protein 1α (MCP-1α) over time (T1–T5) in the overall population (All) and the three groups (OPCAB, CABG on-pump and valve-CABG). Values are presented as median and interquartile range (IQR) (box), within 1.5× IQR (line) and outliers (dots). T1, baseline; T2, time of intensive care unit (ICU) admission; T3, 4 h after surgery; T4, 8 h after surgery; T5, 48 h after surgery; CABG, coronary artery bypass grafting; OPCAB, off-pump coronary artery bypass grafting.

The evolution of IL-6 and MCP-1α over time is displayed in Figure 5. For the overall population, both parameters were present in a minimal concentration at the baseline and were highest at the time of ICU admission. Thereafter, IL-6 decreased progressively without completely reaching the baseline value at 48 h. MCP-1α showed a rapid decline in the first 4 h after surgery and overall tended to decrease more rapidly than IL-6.

The baseline (T1) and time of ICU admission (T2) values of IL-6, MCP-1α, WBC, and CRP are summarized in Table 3. Compared to T1, higher median values were found for IL-6, MCP-1α, and WBC but not for CRP at T2 using Wilcoxon tests for paired data.

Compared to the OPCAB group, very strong evidence was found at the time of ICU admission (T2) for MCP-1α to be, on average, 0.92 ng/ml (0.46–1.38, *p* = 0.0002) higher in the valve-CABG group and moderate evidence of it being, on average, 0.58 ng/ml (0.09–1.07, *p* = 0.0229) higher in the on-pump CABG group. Considering all time points after surgery (T2–T5) in a linear mixed model, strong evidence was found for MCP-1α to be on average 0.27 ng/ml (0.12–0.42, *p* = 0.001) higher in the valve-CABG group than in the OPCAB group with no difference for the on-pump CABG group. For IL-6 no intergroup differences were found.

Between WNT-5a and IL-6, and WNT-5a and MCP-1α no correlation was found at single time points (T1, T2, and T5) nor an association over all time points after surgery (T2–T5).

### Influence of Wingless-related integration site signaling components and inflammatory cytokines on postoperative clinical outcome parameters

For every ng/ml increase of WNT-5a at the time of ICU admission (T2), the fluid balance of the ICU admission day was reduced by −24.81 ml (−49 to −0.46, *p* = 0.046). For every pg/ml increase of WIF-1 at T2, the fluid balance of the ICU admission day increased by 3.81 ml (−0.09 to 7.71, *p* = 0.056), the noradrenaline requirement at the time of ICU admission (T2) increased by 0.017 μg/min (0.00–0.03, *p* = 0.007) and the duration of hemodynamic instability in the ICU increased by 0.9 h (0.03–0.14, *p* = 0.002). For sFRP-5, IL-6 and MCP-1α at T2 no evidence was found for an influence on postoperative hemodynamic outcome parameters.

## Discussion

### Main findings

Temporal changes of WNT-5a, sFRP-1, sFRP-5, and WIF-1 were investigated in the perioperative setting of cardiac surgery. This represents a very standardized and clinically relevant model of sterile inflammation. For the overall population, WNT-5a, sFRP-1, and WIF-1 were present in low concentrations at the baseline, while sFRP-5 was elevated. The baseline values of WNT-5a, sFRP-5, and WIF-1 influenced the evolution of their follow-up values. WNT-5a reached its maximum and sFRP-5 its minimum value at the time of ICU admission with significant changes from the baseline. WNT-5a returned to the baseline within 48 h, while sFRP-5 remained below the baseline at 48 h. Values of sFRP-1 showed a minimal change over time. WIF-1 progressively declined to its minimum value at 8 h after surgery and increased thereafter without reaching the baseline within 48 h. The ratios of WNT-5a/sFRP-5 and WNT-5a/WIF-1 were higher at the time of ICU admission compared to the baseline. Of all investigated WNT signaling components, only WIF-1 partially reflected the severity of surgery at the time of ICU admission. Regarding the inflammatory cytokines, only IL-6 and MCP-1α were strongly expressed with highest values at ICU admission. For MCP-1α statistical differences between the three groups were found at the time of ICU admission. Finally, for WNT-5a and WIF-1 an influence on hemodynamic outcome parameters was found.

### Patient population and clinical outcome

As intended, the three groups differed mainly regarding the complexity of surgery. The duration of postoperative hemodynamic instability and fluid balance after surgery numerically increased with rising surgical complexity across the groups. Accordingly, the valve-CABG-group had the highest noradrenaline dosage at the time of ICU admission. Despite having the highest fluid load intraoperatively, the OPCAB group had the lowest positive fluid balance at the end of the ICU admission day. This could result from less systemic inflammation with lower endothelial permeability compared to the other groups using CPB. The high intraoperative fluid load during OPCAB is explained by a temporary luxation of the heart causing a hemodynamic compromise (23). Considering ICU stay and survival rates, the postoperative course in most patients was favorable. Hence, we were able to compare three homogenous patient populations after cardiac surgery which developed a systemic inflammatory response. This response was sufficiently large to be measured but was not excessive or uncontrolled as clinical outcome was overall benign in our patients.

### Evolution of Wingless-related integration site signaling components upon cardiac surgery

The mononuclear phagocyte system is an essential part of innate immunity and functions as first line defense to tissue damage and microbial infection. Activated macrophages produce WNT-5a and various cytokines that cause an inflammatory response (8). Macrophage activation may be sustained and cytokine production augmented by autocrine binding of WNT-5a to the Frizzled-5 receptor on the macrophage cell membrane (9) and downstream activation of intracellular Ca^2+^/calmodulin-dependent protein kinase 2 (10). Consecutively, WNT-5a signaling in macrophages plays an important role in inflammation because of its potential to upregulate an inflammatory response. However, in-depth knowledge about WNT signaling after cardiac surgery is scarce to date.

In this study, we present baseline plasma concentrations of WNT-5a, sFRP-1, sFRP-5, and WIF-1 in patients with coronary and/or valvular heart disease. These values represent an equilibrium state in a non- or low inflamed patient population as no patient with an active infection or inflammatory disease was included. Little is known about normal reference values of WNT parameters in cardiovascular disease. Elevated basal WNT-5a plasma concentrations have been described in patients with dilated cardiomyopathy and were associated with right ventricular dysfunction, especially in patients with more advanced disease (24). In patients undergoing cardiac surgery, elevated basal WNT-5a values in the serum and epicardial adipose tissue were associated with the presence of coronary artery disease (25). Inter-individual variations of WNT parameter basal plasma concentrations may be relevant for future research. We found very strong evidence that the individual baseline values of WNT-5a, sFRP-5, and WIF-1 had a measurable impact on subsequent values of the respective parameter after surgery. This is of interest, as the interaction between WNT signaling components may considerably influence the inflammatory response (6).

As hypothesized, WNT-5a increased after cardiac surgery but, contrary to our hypothesis, WNT-5a did not reflect the severity of surgery. Howsoever, WNT-5a seems to accurately describe the sterile systemic inflammatory response upon cardiac surgery considering its time-concentration course observed in the overall patient population in this study. It might be assumed that macrophages are maximally activated during surgery and produce a peak WNT-5a concentration to promote inflammation. Accordingly, we found the highest blood plasma concentration of WNT-5a directly after surgery. Thereafter, regulatory mechanisms seem to prevent an uncoupling of WNT-5a signaling from the inflammatory stimulus. In fact, the progressive clearance of WNT-5a within hours after cardiac surgery may indicate the resolution of the inflammatory response in patients with an uncomplicated postoperative course.

A rise of WNT-5a values continuing for hours beyond cardiac surgery may be a reason for concern. In the patient that suffered a perioperative stroke and died on postoperative day 20, we observed an increase of WNT-5a until 8 h after surgery and a return to the baseline value at 48 h. The kinetic profile of WNT-5a in this patient may be the result of an additional inflammatory response to the stroke that occurred perioperatively. Indeed, experimental findings indicate that WNT signaling seems to be critically involved in the pathophysiology of stroke and WNT has been described to rise within hours in brain tissue after ischemic stroke (26). According to our study protocol, no blood sampling occurred between 8 and 48 h after surgery so the time point of the WNT-5a peak concentration in this patient remains at question. Another reason for concern may be the absent recovery of the WNT antagonist sFRP-5 towards the baseline value at 48 h after surgery which was also seen in this patient (data not shown).

Secreted frizzled related proteins (sFRPs) and WIF-1 are considered as soluble antagonists of WNT signaling. They prevent the binding of WNTs to their corresponding cellular receptors by binding themselves to WNT proteins forming a soluble complex that can no longer bind to the WNT receptor. By this way WNT signaling is inhibited by the antagonist (14). In preclinical research, sFRP-1 attenuated a macrophages inflammatory response by preventing the binding of WNT-5a to the membrane bound Frizzled-5 receptor (10). In our study, the sFRP-1 plasma concentration was low at baseline and did not increase after the surgical stimulus. Therefore, we assume that sFRP-1 may not play a relevant role in the sterile inflammatory response after cardiac surgery.

**FIGURE 6:**
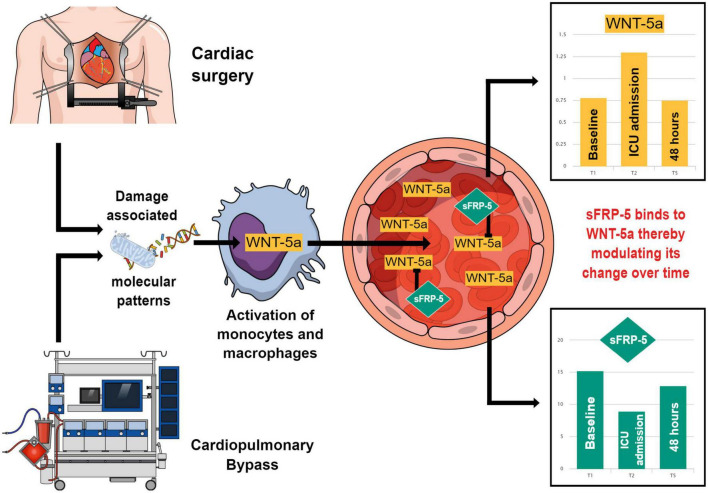
This figure summarizes how Wingless-related integration site 5a (WNT-5a) may come into play in a systemic inflammation caused by cardiac surgery and how its plasma concentration may be modulated by Secreted frizzled-related protein 5 (sFRP-5). Cardiac surgery and the use of cardiopulmonary bypass lead to the formation of damage associated molecular patterns which activate cells of the mononuclear phagocyte system. These cells produce and secrete WNT-5a which enters the circulatory system. In the blood stream, WNT-5a is bound by its antagonist sFRP-5 forming a soluble complex. Between the baseline (T1), the time of intensive care unit (ICU) admission (T2) and 48 h after surgery (T5), the blood plasma concentrations of WNT-5a and sFRP-5 move in the opposite direction compared to each other. This may demonstrate the modulatory effect of sFRP-5 on the WNT-5a plasma concentration leading to an attenuation of the WNT-5a peak value during the promotion of a systemic inflammation upon cardiac surgery and a timely return of WNT-5a to its baseline value during the resolution of the inflammatory response.

For sFRP-5 we found an elevated basal plasma concentration, which is in accordance with a previous study (25). This may indicate that the constitutional level of sFRP-5 is already higher at baseline compared to the baseline level of sFRP-1. The value of sFRP-5 decreased to approximately half of the initial concentration at the time of ICU admission and increased steadily thereafter. This may be due to the formation of a complex between WNT-5a and the soluble antagonist sFRP-5 resulting in a lower plasma concentration of the uncomplexed free form of both parameters. According to preclinical research (14), sFRP-5 may be in fact binding excess levels of WNT-5a and thereby limiting the inflammatory response after cardiac surgery. The effect of cardiac surgery and CPB on the blood plasma concentration of WNT-5a and sFRP-5 and their supposed interaction are graphically presented in Figure 6.

In the experimental setting, WIF-1 antagonized a WNT-5a induced tight junction disruption between VEC by interfering with the Rho-associated protein serine/threonine kinase/LIM kinase 2/Cofilin 1 pathway at the Ryk receptor (11). In our study, WIF-1 was detectable at low concentrations preoperatively and showed a tendency toward lower values until 8 h after surgery. It appears that WIF-1 is constitutionally present at a low plasma concentration and may be depleted from the plasma by complex formation with WNT-5a. Thereby, WIF-1 would prevent the binding of WNT-5a to the Ryk receptor on VEC and limit the extent of microvascular leakage during the inflammatory response after cardiac surgery.

In our study, the kinetic profile of WIF-1 showed some noticeable features in the valve-CABG group. In these patients, WIF-1 at baseline was highest. Furthermore, an intergroup difference was found at the time of ICU admission and the WIF-1 plasma concentration progressively declined until 48 h after surgery. Compared to other patients in this study, patients with combined valvular and coronary heart disease may be in a low inflammatory state. We speculate that basal WIF-1 production might be concomitantly upregulated to maintain a state of equilibrium with WNT-5a. This may explain the higher WIF-1 baseline value and intergroup difference at the time of ICU admission. Our assumption is based on previous findings that WNT-5a was found to be elevated in patients with advanced heart disease (24). It must be noted that the etiology of heart disease may differ considerably between patients with dilated cardiomyopathy and our study population. The decline of WIF-1 beyond 8 h after surgery may result from an ongoing WNT-5a antagonism by WIF-1.

Five measurements within 48 h allowed us to determine the course of the WNT signaling components over time and to extensively investigate their relation to each other. The concentration of WNT-5a evolved in the opposite direction compared to sFRP-5 and WIF-1. Since sFRP-5 and WIF-1 are considered as counterplayers of WNT-5a (14), we calculated the ratios between these parameters to investigate their interaction. The ratios show the balance between the free plasma concentrations of the respective parameters. The increased WNT-5a/sFRP-5 and the WNT-5a/WIF-1 ratios at the time of ICU admission indicate an excess of WNT-5a, which may actively promote the postoperative inflammatory response. Both ratios decreased within 48 h, which was paralleled by the clinical recovery of our patients. The approach of forming a ratio between the agonist and antagonist has been previously used for WNT-5a and sFRP-3 to show the grade of WNT-5a activity in a balanced system (24). Nevertheless, our assumptions on the interaction between WNT signaling components are challenged by the fact that, statistically, no correlation between WNT-5a and sFRP-5 or WIF-1 was found at any given time point in our study.

In this study, a blood product transfusion or steroid stress dose administration did not influence the evolution of WNT-5a, sFRP-5, and WIF-1 after cardiac surgery. In view of the scarcity of evidence on this topic and the small number of patients in our study, this result should be interpreted with caution. To assess the possible effect of an intraoperative blood product transfusion or perioperative hydrocortisone treatment, we included these variables in the mixed models for the WNT signaling components exclusively as a sensitivity analysis. An in-depth investigation of the influence of blood product transfusions or glucocorticoid administration on WNT signaling might be of interest for further research.

### Interaction between Wingless-related integration site-5a and inflammatory cytokines after cardiac surgery

Inflammatory cytokines play a crucial role during a systemic inflammatory response and their kinetic profile in the setting of cardiac surgery has been studied previously (15). In this study, we obtained perioperative kinetic profiles of IL-1β, IL-6, TNF-α, GRO-α, MIP-1α, and MCP-1α. The low baseline values of inflammatory cytokines and more conventional inflammatory markers, CRP and leukocyte count, indicate that our patients were in a low or non-inflammatory state. A presumed upregulation of WNT signaling (particularly in patients with combined valve and coronary artery disease) may be too subtle to induce an upregulation of inflammatory cytokine production.

A particular function of IL-1β is to assist the immune system to defend the host against pathogens (27). In this study, no patient had clinical signs of an infection preoperatively. The low IL-1β values and the kinetic pattern of TNF-α, IL-6, and MCP-1α observed in our patients affirm that the inflammatory response investigated in this study represents a sterile inflammation resulting primarily from cardiac surgery and the use of CPB.

To assess an interaction between WNT-5a and inflammatory cytokines, we decided to use IL-6 and MCP-1α as these parameters have been in use for a long time to demonstrate and explore a systemic inflammation after cardiac surgery (16). WNT-5a, IL-6, and MCP-1α reached a peak concentration at the time of ICU admission and declined thereafter. This finding is in accordance with experimental data at the cellular level where WNT-5a and various cytokines are produced by macrophages upon activation (9). Although we were able to show a temporal relationship between WNT-5a, IL-6, and MCP-1α, no statistical correlation was found between these parameters. Interestingly, we found IL-6 and MCP-1α to increase by a multiple after cardiac surgery compared to WNT-5a barely doubling in plasma concentration. In theory, WNT-5a is considered to amplify the production of inflammatory cytokines in macrophages (9). This may explain why we found a statistical difference for MCP-1α and a trend for IL-6 to reflect the severity of surgery but not for WNT-5a. Our findings are supported by a previous study describing higher blood plasma concentrations of MCP-1 and IL-6 in patients after valve surgery compared to CABG (28). It is WNTs supposed ability to amplify inflammatory cytokine production in macrophages that renders WNT-5a, in our opinion, a valid target to therapeutically influence a systemic inflammation after cardiac surgery by introducing WNT-5a antagonists.

### Influence of Wingless-related integration site signaling components on clinical outcome parameters

One aim of the study was to investigate an impact of WNT signaling components, particularly of WNT-5a, on the postoperative clinical course after cardiac surgery. As clinical surrogates of the inflammatory response we used the postoperative fluid balance and the amount and duration of vasopressor requirement after surgery. Contrary to our study hypothesis, we found an inverse relationship between WNT-5a and WIF-1, and our clinical surrogate parameters for systemic inflammation. However, the associated numerical changes in the fluid balance of the ICU admission day, the noradrenaline requirement at ICU admission and the duration of hemodynamic instability were not in a clinically meaningful range. Referring to experimental data (29), we assumed that WNT-5a would induce a microvascular leakage leading to a higher fluid requirement and WIF-1 would reduce vascular barrier dysfunction resulting in enhanced hemodynamic stability. In the clinical setting, our findings seem to challenge WNT-5a and its associated WNT signaling components as meaningful regulators of an inflammatory response. But it must be stated that each of our three clinical outcome parameters only represent a part of the inflammatory response after cardiac surgery. Consecutively, a composite parameter may more adequately comprise and reflect clinical effects caused by inflammation. Yet, the accurate detection of an inflammation in a patient after cardiac surgery using clinical and laboratory criteria, has been reported as a delicate matter (4).

### Limitations

The results of this study cannot be readily generalized. First, this is a single-center prospective study with a limited sample size leading to a comparably low power, so that significant differences may not have been detected. Second, we included only patients for CABG and valve surgery *via* sternotomy. The WNT signaling components may show a different time-concentration pattern in patients with more extensive cardiac surgery (e.g., type A aortic dissection) or with less tissue trauma if a minimal-invasive approach is used for surgery. Third, no patient in a critical condition, with pre-existing inflammatory disease or with an active infection (e.g., endocarditis) was included in this study. These patients as well may show a different WNT parameter profile after cardiac surgery. Fourth, although no effect of an intraoperative blood product transfusion on WNT signaling was found, this may not apply to patients with a mass transfusion of blood products as no such patient was included in this study. Fifth, WNT signaling components were followed up in this study only until 48 h after surgery. WNT parameter changes beyond this period may have gone unnoticed and, in addition, the point of return of WNT signaling to its preoperative equilibrium state remains uncertain. Sixth, CRP and WBC, two parameters commonly used to characterize an inflammatory state, were only collected at the baseline (T1) and after surgery at the time of ICU admission (T2). It would have been interesting to explore the evolution of CRP and WBC and their interaction with WNT-5a over time but to minimize the amount of blood loss for the patients we decided to include only two time points for these parameters.

## Conclusion

Wingless-related integration site signaling is activated during the inflammatory response after cardiac surgery and its components show relevant plasma concentration changes over time. In patients with an uneventful postoperative course, the WNT-5a plasma concentration rises to a peak value immediately after surgery and returns to the baseline value within 48 h. The time-concentration course of WNT-5a is presumably modulated by the antagonistic action of sFRP-5. Further clinical studies are required to evaluate the significance of WNT-5a as an outcome marker of inflammation and to assess therapeutic options of WNT-5a antagonism to attenuate the effects of a dysregulated inflammatory response in patients after cardiac surgery.

## Data availability statement

The original contributions presented in this study are included in the article/Supplementary material, further inquiries can be directed to the corresponding author.

## Ethics statement

The studies involving human participants were reviewed and approved by the Cantonal Ethics Committee, Zurich, Switzerland (BASEC 2017-01286). The patients/participants provided their written informed consent to participate in this study.

## Author contributions

BK, GH, AR, GHS, DB, GS, and DS: conceptualization and investigation. BK, GH, and GHS: data curation. BK, GH, AR, JB, GS, and DS: formal analysis and methodology. GS and DS: funding acquisition and resources. BK, AR, GHS, GS, and DS: project administration. AR, GS, and DS: supervision. AR, DB, and GS: validation. BK and GH: visualization and writing—original draft. BK, GH, AR, GHS, JB, DB, GS, and DS: writing—review and editing. All authors contributed to the article and approved the submitted version.

## Abbreviations

CABG: coronary artery bypass grafting
CPB: cardiopulmonary bypass
CRP: C-reactive protein
GRO: growth-regulated oncogene
ICU: intensive care unit
IL: interleukin
MCP: monocyte chemoattractant protein
MIP: macrophage inflammatory protein
OPCAB: off-pump coronary aortic bypass
sFRP: secreted frizzled-related protein
TNF: tumor necrosis factor
WBC: white cell blood count
WIF: WNT inhibitory factor
WNT: wingless-related integration site.
