# Supplementary material for: Wingless-related integration site (WNT) signaling is activated during the inflammatory response upon cardiac surgery: A translational study
Source: Front Cardiovasc Med. 2022 Nov 11;9:997350. doi: 10.3389/fcvm.2022.997350 (PMC9692086; doi:10.3389/fcvm.2022.997350)
Supplement: Supplementary file 1 [file Data_Sheet_1.docx]

**Supplementary Table 1.** Blood plasma values of Wingless-related integration site 5a (WNT-5a) at study defined blood sampling time points (T1-T5)

|  | **Total**  **(n = 64)** | **OPCAB**  **(n = 28)** | **On-pump CABG**  **(n = 16)** | **Valve-CABG**  **(n = 20)** |
| --- | --- | --- | --- | --- |
| WNT-5a  (ng/ml)  T1 | 0.78  (0.43‒1.13)^§^  (0.09‒260)^#^ | 0.75  (0.36‒0.98)^§^  (0.09‒260)^#^ | 1.1  (0.76‒2.1)^§^  (0.30‒3.7)^#^ | 0.70  (0.27‒0.90)^§^  (0.09‒62.5)^#^ |
| WNT-5a  (ng/ml)  T2 | 1.3  (0.93‒1.9)^§^  (0.09‒201)^#^ | 1.1  (0.74‒1.3)^§^  (0.09‒201)^#^ | 1.7  (1.4‒2.2)^§^  (1.0‒19.9)^#^ | 1.5  (0.65‒2.3)^§^  (0.09‒12.4)^#^ |
| WNT-5a  (ng/ml)  T3 | 1.1  (0.72‒1.8)^§^  (0.09‒258)^#^ | 1.0  (0.48-1.7)^§^  (0.09‒258)^#^ | 1.5  (1.1‒2.5)^§^  (0.17‒100)^#^ | 1.1  (0.65‒1.7)^§^  (0.09‒6.5)^#^ |
| WNT-5a  (ng/ml)  T4 | 0.93  (0.44‒1.6)^§^  (0.08‒100)^#^ | 0.78  (0.42-1.8)^§^  (0.08‒93.5)^#^ | 1.41  (1.0‒2.5)^§^  (0.11‒100)^#^ | 0.86  (0.27‒1.3)^§^  (0.09‒7.5)^#^ |
| WNT-5a  (ng/ml)  T5 | 0.75  (0.46‒1.06)^§^  (0.09‒22.1)^#^ | 0.69  (0.23‒0.94)^§^  (0.09‒22.1)^#^ | 0.96  (0.78‒1.3)^§^  (0.52‒4.0)^#^ | 0.74  (0.41‒1.2)^§^  (0.09‒7.4)^#^ |

**Legend:** T1, baseline; T2, time of intensive care unit (ICU) admission; T3, 4 h after surgery; T4, 8 h after surgery; T5, 48 h after surgery. Data are presented as median, interquartile range (§) and range (#).

**Supplementary Table 2.** Blood plasma values of Secreted frizzled-related protein 1 (sFRP-1) at study defined blood sampling time points (T1-T5)

|  | **Total**  **(n = 64)** | **OPCAB**  **(n = 28)** | **On-pump**  **CABG**  **(n = 16)** | **Valve-CABG**  **(n = 20)** |
| --- | --- | --- | --- | --- |
| sFRP-1  (ng/ml)  T1 | 0.09  (0.09‒0.48)^§^  (0.09‒99.6)^#^ | 0.09  (0.09‒0.09)^§^  (0.09‒99.6)^#^ | 0.09  (0.09‒1.14)^§^  (0.09‒5.3)^#^ | 0.09  (0.09‒6.23)^§^  (0.09‒66.1)^#^ |
| sFRP-1  (ng/ml)  T2 | 0.09  (0.09‒0.09)^§^  (0.09‒101.8)^#^ | 0.09  (0.09‒0.09)^§^  (0.09‒101.8)^#^ | 0.09  (0.09‒0.17)^§^  (0.09‒30.7)^#^ | 0.09  (0.09‒0.26)^§^  (0.09‒10.8)^#^ |
| sFRP-1  (ng/ml)  T3 | 0.09  (0.09‒0.09)^§^  (0.09‒105)^#^ | 0.09  (0.09‒0.09)^§^  (0.09‒105)^#^ | 0.09  (0.09‒0.10)^§^  (0.09‒4.5)^#^ | 0.09  (0.09‒0.92)^§^  (0.09‒8.8)^#^ |
| sFRP-1  (ng/ml)  T4 | 0.09  (0.09‒0.09)^§^  (0.09‒105)^#^ | 0.09  (0.09‒0.09)^§^  (0.09‒105)^#^ | 0.09  (0.09‒0.09)^§^  (0.09‒3.1)^#^ | 0.09  (0.09‒1.15)^§^  (0.09‒9.3)^#^ |
| sFRP-1  (ng/ml)  T5 | 0.09  (0.09‒0.09)^§^  (0.09‒105)^#^ | 0.09  (0.09‒0.09)^§^  (0.09‒105)^#^ | 0.09  (0.09‒0.36)^§^  (0.09‒7)^#^ | 0.09  (0.09‒0.45)^§^  (0.09‒13.8)^#^ |

**Legend:** T1, baseline; T2, time of intensive care unit (ICU) admission; T3, 4 h after surgery; T4, 8 h after surgery; T5, 48 h after surgery. Data are presented as median, interquartile range (§) and range (#).

**Supplementary Table 3.** Blood plasma values of Secreted frizzled-related protein 5 (sFRP-5) at study defined blood sampling time points (T1-T5)

|  | **Total**  **(n = 64)** | **OPCAB**  **(n = 28)** | **On-pump**  **CABG**  **(n = 16)** | **Valve-CABG**  **(n = 20)** |
| --- | --- | --- | --- | --- |
| sFRP-5  (ng/ml)  T1 | 15.2  (10.3‒23.6)^§^  (0.64‒89.3)^#^ | 14.2  (9.7‒18.2)^§^  (0.64‒56.4)^#^ | 21.3  (13.0‒31.0)^§^  (0.64‒55.6)^#^ | 14.6  (9.6‒23.1)^§^  (5.09‒89.3)^#^ |
| sFRP-5  (ng/ml)  T2 | 8.9  (3.8‒12.8)^§^  (0.64‒52.4)^#^ | 8.9  (4.1‒11.6)^§^  (0.64‒52.4)^#^ | 8.0  (0.6‒17.3)^§^  (0.64‒38)^#^ | 9.6  (5.5‒12.7)^§^  (0.64‒45.9)^#^ |
| sFRP-5  (ng/ml)  T3 | 9.1  (4.2‒14.3)^§^  (0.64‒45.7)^#^ | 8.9  (3.4‒13.5)^§^  (0.64‒45.7)^#^ | 9.7  (0.6‒17.0)^§^  (0.64‒45.2)^#^ | 8.6  (4.8‒15.5)^§^  (0.64‒43.7)^#^ |
| sFRP-5  (ng/ml)  T4 | 9.7  (5.0‒16.0)^§^  (0.64‒46.9)^#^ | 9.7  (5.0‒12.4)^§^  (0.64‒46.9)^#^ | 9.7  (0.6‒24.4)^§^  (0.64‒43.2)^#^ | 9.7  (6.2‒16.5)^§^  (0.64‒38.5)^#^ |
| sFRP-5  (ng/ml)  T5 | 12.8  (7.9‒17.2)^§^  (0.64‒235)^#^ | 11.8  (7.5‒16.3)^§^  (0.64‒33.6)^#^ | 14.3  (7.6‒19.1)^§^  (0.64‒39.6)^#^ | 12.8  (9.3‒20.2)^§^  (0.64‒235)^#^ |

**Legend:** T1, baseline; T2, intensive care unit (ICU) admission; T3, 4 h after surgery; T4, 8 h after surgery; T5, 48 h after surgery. Data are presented as median, interquartile range (§) and range (#).

**Supplementary Table 4.** Blood plasma values of WNT inhibitory factor 1 (WIF-1) at study defined blood sampling time points (T1-T5)

|  | **Total**  **(n = 64)** | **OPCAB**  **(n = 28)** | **On-pump**  **CABG**  **(n = 16)** | **Valve-CABG**  **(n = 20)** |
| --- | --- | --- | --- | --- |
| WIF-1  (ng/ml)  T1 | 0.17  (0.08‒0.28)^§^  (0.01‒0.68)^#^ | 0.12  (0.06‒0.21)^§^  (0.01‒0.68)^#^ | 0.19  (0.08‒0.29)^§^  (0.01‒0.68)^#^ | 0.19  (0.12‒0.41)^§^  (0.01‒0.61)^#^ |
| WIF-1  (ng/ml)  T2 | 0.13  (0.09‒0.3)^§^  (0.01‒0.85)^#^ | 0.1  (0.09‒0.18)^§^  (0.01‒0.56)^#^ | 0.13  (0.03‒0.39)^§^  (0.01‒0.6)^#^ | 0.18  (0.13‒0.44)^§^  (0.01‒0.85)^#^ |
| WIF-1  (ng/ml)  T3 | 0.13  (0.06‒0.3)^§^  (0.01‒0.68)^#^ | 0.09  (0.05‒0.2)^§^  (0.01‒0.49)^#^ | 0.11  (0.01‒0.36)^§^  (0.01‒0.64)^#^ | 0.16  (0.1‒0.4)^§^  (0.01‒0.68)^#^ |
| WIF-1  (ng/ml)  T4 | 0.12  (0.07‒0.28)^§^  (0.01‒0.68)^#^ | 0.08  (0.03‒0.15)^§^  (0.01‒0.58)^#^ | 0.15  (0.05‒0.35)^§^  (0.01‒0.66)^#^ | 0.14  (0.11‒0.41)^§^  (0.01‒0.68)^#^ |
| WIF-1  (ng/ml)  T5 | 0.13  (0.07‒0.31)^§^  (0.01‒0.59)^#^ | 0.12  (0.06‒0.17)^§^  (0.01‒0.47)^#^ | 0.16  (0.05‒0.36)^§^  (0.01‒0.59)^#^ | 0.13  (0.09‒0.38)^§^  (0.01‒0.57)^#^ |

**Legend:** T1, baseline; T2, time of intensive care unit (ICU) admission; T3, 4 h after surgery; T4, 8 h after surgery; T5, 48 h after surgery. Data are presented as median, interquartile range (§) and range (#).

**Supplementary Table 5.** Blood plasma values of ratio WNT-5a/sFRP-5 at study defined blood sampling time points (T1-T5)

|  | **Total**  **(n = 64)** | **OPCAB**  **(n = 28)** | **On-pump**  **CABG**  **(n = 16)** | **Valve-CABG**  **(n = 20)** |
| --- | --- | --- | --- | --- |
| WNT-5a/sFRP-5  T1 | 0.04  (0.02‒0.07)^§^  (0.00‒12)^#^ | 0.04  (0.02‒0.08)^§^  (0.01‒9.3)^#^ | 0.05  (0.03‒0.1)^§^  (0.02‒1.6)^#^ | 0.03  (0.01‒0.06)^§^  (0.00‒12)^#^ |
| WNT-5a/sFRP-5  T2 | 0.14  (0.1‒0.26)^§^  (0.02‒31)^#^ | 0.13  (0.08‒0.17)^§^  (0.02‒14)^#^ | 0.19  (0.14‒1.88)^§^  (0.03‒31)^#^ | 0.15  (0.09‒0.23)^§^  (0.02‒19)^#^ |
| WNT-5a/sFRP-5  T3 | 0.13  (0.06‒0.21)^§^  (0.01‒156)^#^ | 0.11  (0.06‒0.18)^§^  (0.01‒11)^#^ | 0.13  (0.1‒1.17)^§^  (0.02‒156)^#^ | 0.11  (0.04‒0.21)^§^  (0.02‒10)^#^ |
| WNT-5a/sFRP-5  T4 | 0.08  (0.04‒0.16)^§^  (0.01‒156)^#^ | 0.08  (0.03‒0.17)^§^  (0.01‒5)^#^ | 0.1  (0.06‒1.3)^§^  (0.02‒156)^#^ | 0.06  (0.03‒0.12)^§^  (0.01‒12)^#^ |
| WNT-5a/sFRP-5  T5 | 0.06  (0.03‒0.15)^§^  (0.00‒12)^#^ | 0.06  (0.03‒0.16)^§^  (0.00‒4.4)^#^ | 0.12  (0.04‒1.3)^§^  (0.01‒6.64)^#^ | 0.05  (0.03‒0.08)^§^  (0.00‒12)^#^ |

**Legend:** sFRP, Secreted frizzled-related protein; WNT, Wingless-related integration site; T1, baseline; T2, time of intensive care unit (ICU) admission; T3, 4 h after surgery; T4, 8 h after surgery; T5, 48 h after surgery. Data are presented as median, interquartile range (§) and range (#).

**Supplementary Table 6.** Blood plasma values of ratio WNT-5a/WIF-1 at study defined blood sampling time points (T1-T5)

|  | **Total**  **(n = 64)** | **OPCAB**  **(n = 28)** | **On-pump**  **CABG**  **(n = 16)** | **Valve-CABG**  **(n = 20)** |
| --- | --- | --- | --- | --- |
| WNT-5a/WIF-1  T1 | 3.4  (1.5‒18.2)^§^  (0.45‒10601)^#^ | 5.2  (1.7‒34.1)^§^  (0.45‒735)^#^ | 4.9  (3.1‒33.4)^§^  (1.1‒613)^#^ | 1.8  (1.5‒3.3)^§^  (0.49‒10601)^#^ |
| WNT-5a/WIF-1  T2 | 8.42  (3.2‒22.6)^§^  (0.69‒3374)^#^ | 10.5  (3.0‒23)^§^  (0.69‒454)^#^ | 15  (4.44‒83)^§^  (2.1‒3374)^#^ | 4.27  (2.9‒8.8)^§^  (0.71‒2101)^#^ |
| WNT-5a/WIF-1  T3 | 5.9  (2.6‒26.8)^§^  (0.46‒16949)^#^ | 9.5  (2.5‒48)^§^  (0.46‒522)^#^ | 16.4  (3.25‒127)^§^  (1.12‒16949)^#^ | 3.9  (2.49‒7.52)^§^  (0.49‒1098)^#^ |
| WNT-5a/WIF-1  T4 | 4.3  (2.17‒15.9)^§^  (0.63‒16949)^#^ | 6.3  (1.82‒77)^§^  (0.63‒396)^#^ | 6.9  (2.8‒85)^§^  (0.68‒16949)^#^ | 2.6  (1.8‒4.9)^§^  (0.73‒1269)^#^ |
| WNT-5a/WIF-1  T5 | 5.0  (2.3‒14.4)^§^  (0.45‒16949)^#^ | 6.2  (1.8‒19)^§^  (0.49‒380)^#^ | 9.1  (3.1‒32)^§^  (0.45‒16949)^#^ | 3.5  (2.2‒5.9)^§^  (0.7‒1269)^#^ |

**Legend:** WIF, WNT inhibitory factor; WNT, Wingless-related integration site; T1, baseline; T2, time of intensive care unit (ICU) admission; T3, 4 h after surgery; T4, 8 h after surgery; T5, 48 h after surgery. Data are presented as median, interquartile range (§) and range (#).

**Supplementary Table 7.** Blood plasma values of tumor necrosis factor α (TNF-α) at study defined blood sampling time points (T1-T5)

|  | **Total**  **(n = 64)** | **OPCAB**  **(n = 28)** | **On-pump**  **CABG**  **(n = 16)** | **Valve-CABG**  **(n = 20)** |
| --- | --- | --- | --- | --- |
| TNF-α  (ng/ml)  T1 | 0.01  (0.00‒0.03)^§^  (0.00‒0.05)^#^ | 0.01  (0.00‒0.02)^§^  (0.00‒0.04)^#^ | 0.01  (0.00‒0.03)^§^  (0.00‒0.05)^#^ | 0.01  (0.00‒0.03)^§^  (0.00‒0.04)^#^ |
| TNF-α  (ng/ml)  T2 | 0.04  (0.03‒0.06)^§^  (0.00‒0.28)^#^ | 0.03  (0.02‒0.04)^§^  (0.0‒0.09)^#^ | 0.04  (0.03‒0.11)^§^  (0.0‒0.28)^#^ | 0.06  (0.04‒0.07)^§^  (0.02‒0.1)^#^ |
| TNF-α  (ng/ml)  T3 | 0.03  (0.02‒0.05)^§^  (0.0‒0.13)^#^ | 0.02  (0.02‒0.04)^§^  (0.0‒0.06)^#^ | 0.03  (0.01‒0.05)^§^  (0.0‒0.13)^#^ | 0.04  (0.03‒0.06)^§^  (0.01‒0.09)^#^ |
| TNF-α  (ng/ml)  T4 | 0.02  (0.01‒0.03)^§^  (0.0‒0.14)^#^ | 0.02  (0.001‒-0.02)^§^  (0.0‒0.05)^#^ | 0.02  (0.01‒0.04)^§^  (0.0‒0.1)^#^ | 0.03  (0.02‒0.04)^§^  (0.0‒0.14)^#^ |
| TNF-α  (ng/ml)  T5 | 0.01  (0.00‒0.02)^§^  (0.00‒0.1)^#^ | 0.01  (0.002‒0.02)^§^  (0.00‒0.09)^#^ | 0.02  (0.00‒0.02)^§^  (0.00‒0.04)^#^ | 0.02  (0.01‒0.03)^§^  (0.00‒0.10)^#^ |

**Legend:** T1, baseline; T2, time of intensive care unit (ICU) admission; T3, 4 h after surgery; T4, 8 h after surgery; T5, 48 h after surgery. Data are presented as median, interquartile range (§) and range (#).

**Supplementary Table 8.** Blood plasma values of interleukin-6 (IL-6) at study defined blood sampling time points (T1-T5)

|  | **Total**  **(n = 64)** | **OPCAB**  **(n = 28)** | **On-pump**  **CABG**  **(n = 16)** | **Valve-CABG**  **(n = 20)** |
| --- | --- | --- | --- | --- |
| IL-6  (ng/ml)  T1 | 0.00  (0.00‒0.00)^§^  (0.00‒0.02)^#^ | 0.00  (0.00‒0.00)^§^  (0.00‒0.02)^#^ | 0.00  (0.00‒0.00)^§^  (0.00‒0.00)^#^ | 0.00  (0.00‒0.00)^§^  (0.00‒0.00)^#^ |
| IL-6  (ng/ml)  T2 | 0.28  (0.14‒0.66)^§^  (0.01‒934)^#^ | 0.15  (0.11‒0.33)^§^  (0.01‒0.72)^#^ | 0.29  (0.15‒0.40)^§^  (0.06‒1.03)^#^ | 0.76  (0.33‒1.30)^§^  (0.07‒934)^#^ |
| IL-6  (ng/ml)  T3 | 0.25  (0.16‒0.46)^§^  (0.01‒1.73)^#^ | 0.22  (0.16‒0.47)^§^  (0.01‒1.25)^#^ | 0.18  (0.14‒0.39)^§^  (0.08‒0.53)^#^ | 0.31  (0.22‒0.47)^§^  (0.06‒1.73)^#^ |
| IL-6  (ng/ml)  T4 | 0.18  (0.11‒0.26)^§^  (0.01‒1.21)^#^ | 0.17  (0.10‒0.27)^§^  (0.01‒0.66)^#^ | 0.19  (0.11‒0.22)^§^  (0.05‒0.45)^#^ | 0.19  (0.11‒0.27)^§^  (0.05‒1.21)^#^ |
| IL-6  (ng/ml)  T5 | 0.03  (0.02‒0.05)^§^  (0.00‒0.19)^#^ | 0.03  (0.02‒0.05)^§^  (0.00‒0.19)^#^ | 0.03  (0.02‒0.04)^§^  (0.01‒0.1)^#^ | 0.03  (0.02‒0.05)^§^  (0.00‒0.12)^#^ |

**Legend:** T1, baseline; T2, time of intensive care unit (ICU) admission; T3, 4 h after surgery; T4, 8 h after surgery; T5, 48 h after surgery. Data are presented as median, interquartile range (§) and range (#).

**Supplementary Table 9.** Blood plasma values of monocyte chemoattractant protein 1α (MCP-1α) at study defined blood sampling time points (T1-T5)

|  | **Total**  **(n = 64)** | **OPCAB**  **(n = 28)** | **On-pump**  **CABG**  **(n = 16)** | **Valve-CABG**  **(n = 20)** |
| --- | --- | --- | --- | --- |
| MCP-1α  (ng/ml)  T1 | 0.04  (0.02‒0.05)^§^  (0.01‒0.15)^#^ | 0.03  (0.02‒0.05)^§^  (0.01‒0.08)^#^ | 0.03  (0.02‒0.04)^§^  (0.02‒0.15)^#^ | 0.04  (0.03‒0.05)^§^  (0.02‒0.06)^#^ |
| MCP-1α  (ng/ml)  T2 | 0.50  (0.28‒1.05)^§^  (0.04‒4.2)^#^ | 0.34  (0.21‒0.64)^§^  (0.04‒1.1)^#^ | 0.55  (0.28‒1.16)^§^  (0.1‒3.34)^#^ | 1.10  (0.74‒1.49)^§^  (0.31‒4.2)^#^ |
| MCP-1α  (ng/ml)  T3 | 0.19  (0.11‒0.28)^§^  (0.03‒1.02)^#^ | 0.20  (0.10‒0.29)^§^  (0.03‒0.96)^#^ | 0.15  (0.10‒0.23)^§^  (0.05‒0.85)^#^ | 0.19  (0.11‒0.27)^§^  (0.07‒1.02)^#^ |
| MCP-1α  (ng/ml)  T4 | 0.08  (0.06‒0.13)^§^  (0.00‒1.37)^#^ | 0.08  (0.05‒0.11)^§^  (0.00‒0.4)^#^ | 0.08  (0.06‒0.11)^§^  (0.03‒0.44)^#^ | 0.09  (0.07‒0.15)^§^  (0.03‒1.37)^#^ |
| MCP-1α  (ng/ml)  T5 | 0.05  (0.04‒0.07)^§^  (0.02‒0.83)^#^ | 0.05  (0.04‒0.06)^§^  (0.02‒0.19)^#^ | 0.05  (0.03‒0.07)^§^  (0.02‒0.17)^#^ | 0.05  (0.03‒0.09)^§^  (0.02‒0.83)^#^ |

**Legend:** T1, baseline; T2, time of intensive care unit (ICU) admission; T3, 4 h after surgery; T4, 8 h after surgery; T5, 48 h after surgery. Data are presented as median, interquartile range (§) and range (#).

**Supplementary Table 10.** Blood plasma values of growth-regulated oncogene α (GRO-α) at study defined blood sampling time points (T1-T5)

|  | **Total**  **(n = 64)** | **OPCAB**  **(n = 28)** | **On-pump**  **CABG**  **(n = 16)** | **Valve-CABG**  **(n = 20)** |
| --- | --- | --- | --- | --- |
| GRO-α  (ng/ml)  T1 | 0.01  (0.01‒0.03)^§^  (0.01‒0.38)^#^ | 0.01  (0.01‒0.01)^§^  (0.01‒0.38)^#^ | 0.01  (0.01‒0.03)^§^  (0.01‒0.16)^#^ | 0.01  (0.01‒0.09)^§^  (0.01‒0.15)^#^ |
| GRO-α  (ng/ml)  T2 | 0.24  (0.13‒0.35)^§^  (0.01‒0.86)^#^ | 0.15  (0.08‒0.21)^§^  (0.01‒0.53)^#^ | 0.27  (0.09‒0.45)^§^  (0.01‒0.86)^#^ | 0.32  (0.27‒0.42)^§^  (0.15‒0.67)^#^ |
| GRO-α  (ng/ml)  T3 | 0.09  (0.01‒0.20)^§^  (0.00‒0.59)^#^ | 0.03  (0.01‒0.10)^§^  (0.0‒0.57)^#^ | 0.08  (0.01‒0.22)^§^  (0.01‒0.59)^#^ | 0.18  (0.11‒0.25)^§^  (0.01‒0.47)^#^ |
| GRO-α  (ng/ml)  T4 | 0.05  (0.01‒0.14)^§^  (0.01‒0.37)^#^ | 0.01  (0.01‒0.02)^§^  (0.01‒0.28)^#^ | 0.07  (0.01‒0.16)^§^  (0.01‒0.37)^#^ | 0.14  (0.05‒0.19)^§^  (0.01‒0.35)^#^ |
| GRO-α  (ng/ml)  T5 | 0.01  (0.01‒0.06)^§^  (0.01‒0.24)^#^  n=63 | 0.01  (0.01‒0.01)^§^  (0.01‒0.24)^#^ | 0.01  (0.01‒0.01)^§^  (0.01‒0.13)^#^ | 0.01  (0.01‒0.09)^§^  (0.01‒0.23)^#^  n=19 |

**Legend:** T1, baseline; T2, time of intensive care unit (ICU) admission; T3, 4 h after surgery; T4, 8 h after surgery; T5, 48 h after surgery. Data are presented as median, interquartile range (§) and range (#).

**Supplementary Table 11.** Blood plasma values of macrophage inflammatory protein 1α (MIP-1α) at study defined blood sampling time points (T1-T5)

|  | **Total**  **(n = 64)** | **OPCAB**  **(n = 28)** | **On-pump**  **CABG**  **(n = 16)** | **Valve-CABG**  **(n = 20)** |
| --- | --- | --- | --- | --- |
| MIP-1α  (ng/ml)  T1 | 0.00  (0.00‒0.00)^§^  (0.00‒0.06)^#^ | 0.00  (0.00‒0.00)^§^  (0.00‒0.06)^#^ | 0.00  (0.00‒0.00)^§^  (0.00‒0.01)^#^ | 0.00  (0.00‒0.00)^§^  (0.00‒0.02)^#^ |
| MIP-1α  (ng/ml)  T2 | 0.02  (0.01‒0.04)^§^  (0.00‒0.23)^#^ | 0.01  (0.01‒0.02)^§^  (0.00‒0.1)^#^ | 0.02  (0.02‒0.04)^§^  (0.00‒0.23)^#^ | 0.04  (0.03‒0.05)^§^  (0.01‒0.14)^#^ |
| MIP-1α  (ng/ml)  T3 | 0.01  (0.00‒0.02)^§^  (0.00‒0.15)^#^ | 0.01  (0.003‒0.01)^§^  (0.00‒0.15)^#^ | 0.01  (0.01‒0.01)^§^  (0.00‒0.09)^#^ | 0.02  (0.01‒0.02)^§^  (0.01‒0.06)^#^ |
| MIP-1α  (ng/ml)  T4 | 0.01  (0.00‒0.01)^§^  (0.00‒0.08)^#^ | 0.00  (0.001‒0.005)^§^  (0.00‒0.08)^#^ | 0.01  (0.004‒0.01)^§^  (0.00‒0.04)^#^ | 0.01  (0.01‒0.01)^§^  (0.00‒0.04)^#^ |
| MIP-1α  (ng/ml)  T5 | 0.00  (0.00‒0.00)^§^  (0.00‒0.06)^#^  n=63 | 0.00  (0.00‒0.00)^§^  (0.00‒0.06)^#^ | 0.00  (0.00‒0.00)^§^  (0.00‒0.00)^#^ | 0.00  (0.00‒0.00)^§^  (0.00‒0.01)^#^  n=19 |

**Legend:** T1, baseline; T2, intensive care unit (ICU) admission; T3, 4 h after surgery; T4, 8 h after surgery; T5, 48 h after surgery. Data are presented as median, interquartile range (§) and range (#).

**Supplementary Figure 1.**


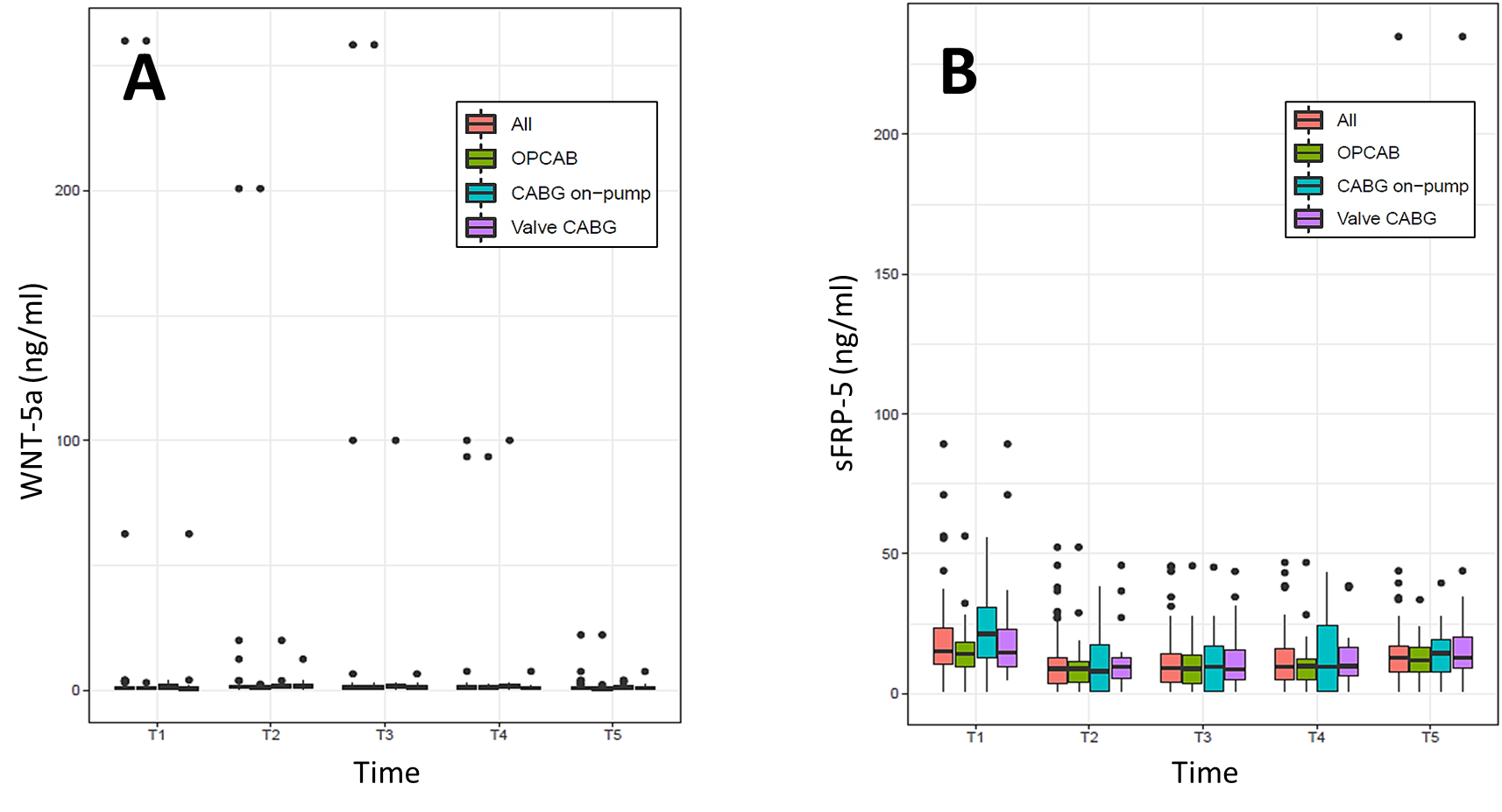


**Legend:** Evolution of (**A**) Wingless-related integration site 5a (WNT-5a) and (**B**) Secreted frizzled-related protein 5 (sFRP-5) over time (T1-T5) in the overall population and the three groups (OPCAB, CABG on-pump and valve-CABG). Values are presented as median and interquartile range (IQR) (box), within 1.5x IQR (line) and outliers (dots). T1, baseline; T2, time of intensive care unit (ICU) admission; T3, 4 h after surgery; T4, 8 h after surgery; T5, 48 h after surgery; CABG, coronary artery bypass grafting; OPCAB, off-pump coronary artery bypass grafting.

**Supplementary Figure 2.**


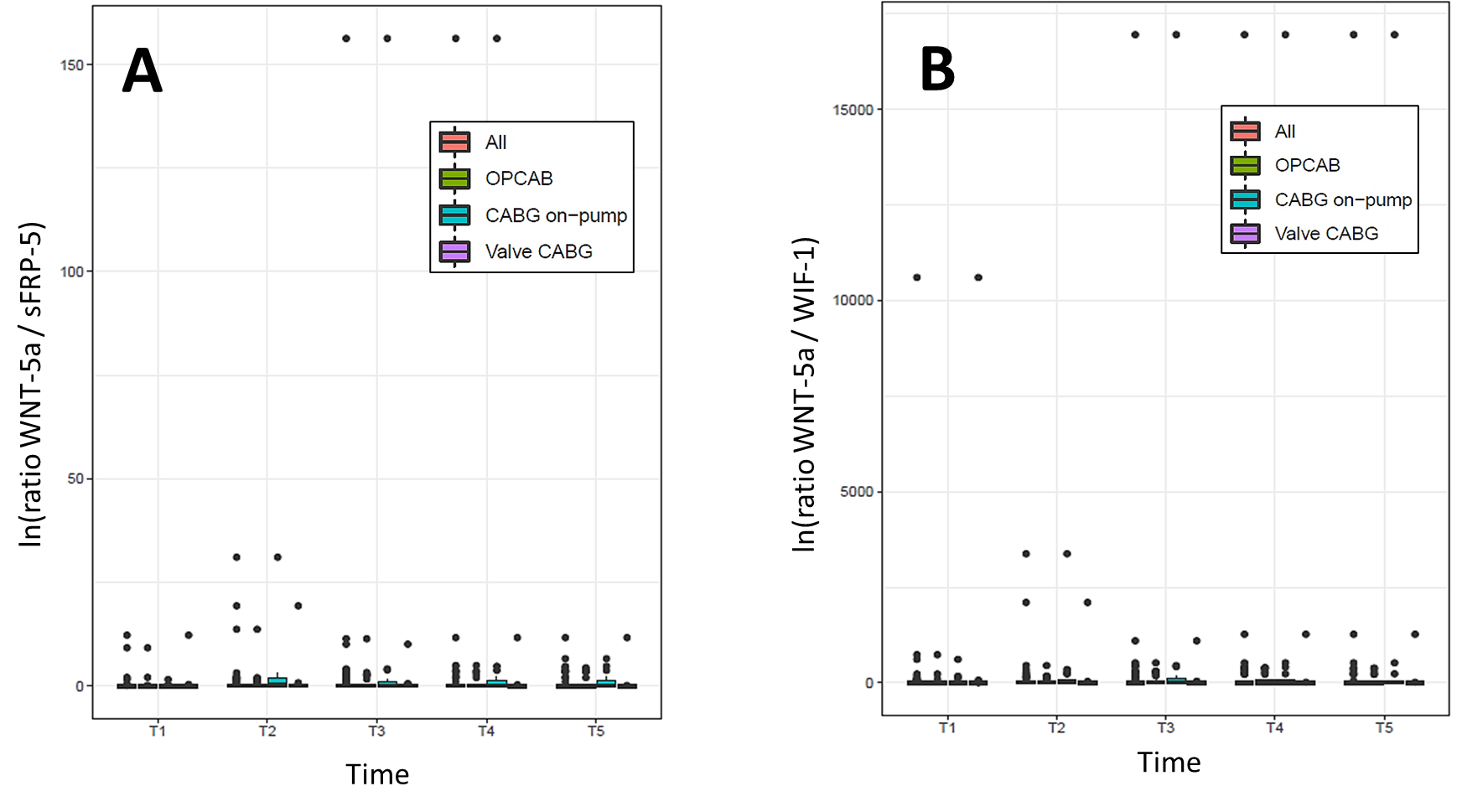


**Legend:** Evolution of ratios between (**A**) Wingless-related integration site 5a (WNT-5a) and Secreted frizzled-related protein 5 (sFRP-5), and (**B**) between WNT-5a and WNT inhibitory factor 1 (WIF-1) over time (T1-T5) in the overall population and the three groups (OPCAB, CABG on-pump and valve-CABG). Values are presented as median and interquartile range (IQR) (box), within 1.5x IQR (line) and outliers (dots). T1, baseline; T2, time of intensive care unit (ICU) admission; T3, 4 h after surgery; T4, 8 h after surgery; T5, 48 h after surgery; CABG, coronary artery bypass grafting; OPCAB, off-pump coronary artery bypass grafting.

**Supplementary Figure 3.**


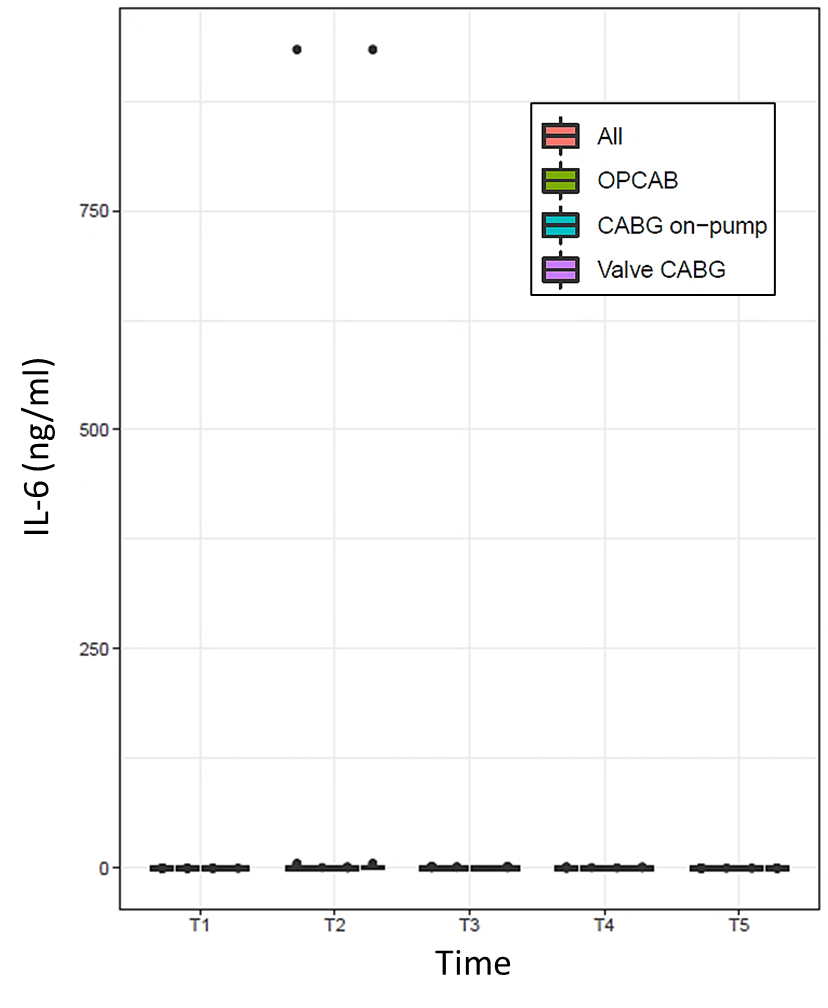


**Legend:** Evolution of interleukin-6 (IL-6) over time (T1-T5) in the overall population and the three groups (OPCAB, CABG on-pump and valve-CABG). Values are presented as median and interquartile range (IQR) (box), within 1.5x IQR (line) and outliers (dots). T1, baseline; T2, time of intensive care unit (ICU) admission; T3, 4 h after surgery; T4, 8 h after surgery; T5, 48 h after surgery; CABG, coronary artery bypass grafting; OPCAB, off-pump coronary artery bypass grafting.
